# TatBC-Independent TatA/Tat Substrate Interactions Contribute to Transport Efficiency

**DOI:** 10.1371/journal.pone.0119761

**Published:** 2015-03-16

**Authors:** Johannes Taubert, Bo Hou, H. Jelger Risselada, Denise Mehner, Heinrich Lünsdorf, Helmut Grubmüller, Thomas Brüser

**Affiliations:** 1 Institute of Microbiology, Leibniz Universität Hannover, Schneiderberg 50, 30167, Hannover, Germany; 2 Max Planck Institute for Biophysical Chemistry, Am Fassberg 11, 37077, Göttingen, Germany; 3 Helmholtz Centre of Infection Research, Inhoffenstraße 7, 38124, Braunschweig, Germany; Centre National de la Recherche Scientifique, Aix-Marseille Université, FRANCE

## Abstract

The Tat system can transport folded, signal peptide-containing proteins (Tat substrates) across energized membranes of prokaryotes and plant plastids. A twin-arginine motif in the signal peptide of Tat substrates is recognized by TatC-containing complexes, and TatA permits the membrane passage. Often, as in the model Tat systems of *Escherichia coli* and plant plastids, a third component – TatB – is involved that resembles TatA but has a higher affinity to TatC. It is not known why most TatA dissociates from TatBC complexes *in vivo* and distributes more evenly in the membrane. Here we show a TatBC-independent substrate-binding to TatA from *Escherichia coli*, and we provide evidence that this binding enhances Tat transport. First hints came from *in vivo* cross-linking data, which could be confirmed by affinity co-purification of TatA with the natural Tat substrates HiPIP and NrfC. Two positions on the surface of HiPIP could be identified that are important for the TatA interaction and transport efficiency, indicating physiological relevance of the interaction. Distributed TatA thus may serve to accompany membrane-interacting Tat substrates to the few TatBC spots in the cells.

## Introduction

The twin-arginine translocation (Tat) system is a general translocation system that serves to translocate folded proteins with N-terminal signal peptides across energized membranes in prokaryotes and plant plastids [1]. Tat signal peptides contain a characteristic amino acid pattern that typically includes the two eponymous arginines [2]. Tat systems minimally consist of the two components TatA and TatC [3]. Often a third component that is sequence-related to TatA is found that has a higher affinity to TatC and that is termed TatB [2]. In *E*. *coli* as well as in plant plastids, TatBC-containing complexes recognize the twin-arginine motif and thereby bind the Tat substrates [4,5,6,7]. Some TatA associates with TatBC complexes already before substrate-binding and can be important for high-affinity binding of substrates at physiological conditions [8,9], but additional TatA is recruited or re-organized at the translocon upon substrate-binding [10] and plays an important role when the folded part is transferred through the membrane [10,11,12,13,14,15]. However, most TatA forms homo-oligomeric associations distributed in the membrane, whereas the active translocons are organized in few spots per cell [16,17,18,19]. TatA is a small (usually <10 kDa) membrane protein with a simple topology: a single N-terminal short membrane anchor, followed by an amphipathic helix and a highly charged and most likely largely unstructured C-terminus. The N-terminal membrane anchor and the amphipathic helix are connected by a hinge that contains a highly conserved FG motif. Near the end of the amphipathic helix, an equally conserved FK motif exists [2]. Deep in the lipid bilayer, a contact of the trans-membrane domain of TatA with Tat substrates has been demonstrated to depend on TatBC [20], suggesting that Tat substrates contact the membrane-embedded part of TatA late in transport. In agreement with this finding, the trans-membrane domain of TatA is proposed to allow the passage of the substrate by destabilizing the membrane [11,15]. Recent data showed that recombinant production of Tat substrates results in a recruitment of TatA to the active translocon sites, and the co-localization of TatA at active translocons depends on twin-arginine motif recognition [19]. It would be important to know whether individual membrane-interacting Tat substrates first associate with TatA before they encounter TatBC complexes, as such an interaction could attribute a role to the freely diffusing TatA oligomers. Such a function has been suggested for *Streptomyces* and *Bacillus* species [21,22,23], but since no TatBC-independent TatA/Tat substrate interactions were recognized in the model systems *E*. *coli* and plant plastids, these reports were so far not taken as evidence for a general TatA functionality.

Here we show *in vivo* and *in vitro* that *E*. *coli* TatA can bind Tat substrates. This interaction requires the signal peptide in conjunction with determinants of the mature domain surface and appears to be physiologically relevant for efficient transport of various Tat substrates, attributing a targeting function to the freely diffusing TatA oligomers.

## Results

### TatBC-independent TatA/Tat substrate interactions

We addressed the substrate-translocon interactions of the Tat system by a site-directed *in vivo* cross-linking method that has been developed by the group of Peter G. Schultz [24,25]. In this method, a specific amber stop codon suppressor tRNA is loaded exclusively with the artificial amino acid *p*-benzoyl-L-phenylalanine (*p*Bpa), which is efficiently incorporated into engineered amber stop codons of recombinant proteins. Irradiation of the living cells with UV light of 365 nm then activates the *p*Bpa residue that forms a covalent bond to molecules in its immediate environment. We introduced *p*Bpa into selected positions of the model Tat substrate HiPIP from *Allochromatium vinosum*, which is exclusively Tat dependently translocated in *E*. *coli* [26], and used the low copy vector pRK-*tatABC* to express the *tatABC* operon ∼15-fold from its natural promoter [17]. After *in vivo* photo cross-linking, the membranes were prepared and SDS-solubilized, and HiPIP (that was produced with a C-terminal His_6_-tag) was affinity purified under denaturing conditions. Cross-links of the signal peptide to Tat system components resulted in shifts in SDS-PAGE analyses that were detected using specific antibodies after Western-blotting. A probe of the solubilized membrane sample prior to affinity chromatography was used to compare cross-link efficiencies. UV-induced cross-links eluted in the second and third elution fraction (S1 Fig.). As the method involves a purification step under denaturing conditions, any shifted bands are covalent cross-links that are due to photo-activated *p*Bpa, and, as expected, minus UV-controls showed no cross-links (S1 Fig. shows minus UV-controls for the strongest cross-link position, M26*p*Bpa, and the most intensively used position in this study, A13*p*Bpa). Similarly, wt HiPIP did not cross-link to any wt Tat component in the presence of *p*Bpa and the pEvol-system, as *p*Bpa certainly cannot be incorporated into HiPIP or Tat components when the corresponding genes lack TAG stop codon positions (S1 Fig.).

**Fig 1:**
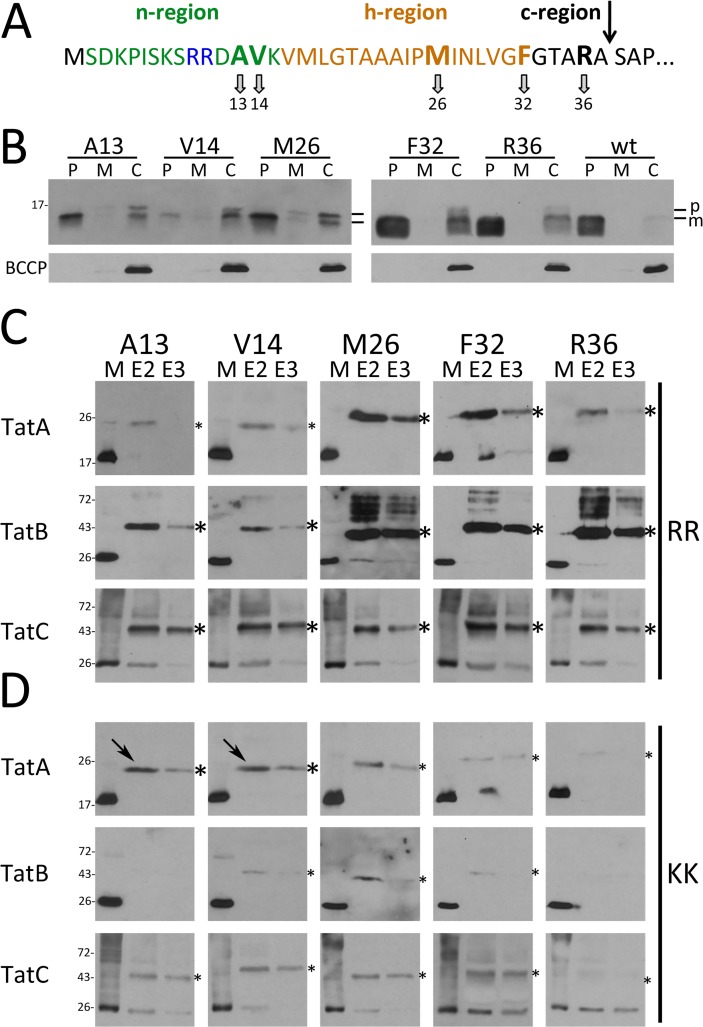
*In vivo* site-directed cross-linking of Tat signal peptides to the TatABC components. (A) Amino acid sequence and positions of the *p*Bpa cross-linker in the Tat signal peptide. The arrow indicates the signal peptide cleavage site. (B) Transport of native HiPIP and variants (produced from pEXH5-tac-H6 derivatives) with *p*Bpa exchanges at indicated positions in pEvol-pBpF/pRK-*tatABC*-containing strains grown in the presence of 0.1 mM *p*Bpa. Detection of HiPIP in subcellular fractions by SDS-PAGE/Western-blotting; the cytoplasmic biotin carboxyl carrier protein (BCCP; ∼22 kDa) was detected to control periplasmic purity. P: periplasm; M: membrane; C: cytoplasm; p: precursor HiPIP; m: mature HiPIP. (C) *In vivo* cross-links of twin-arginine signal peptides to Tat translocon components with *p*Bpa at indicated positions. Specific cross-links are detected in affinity-chromatography elution fractions E2 and E3 by Western blotting using indicated Tat component-specific antibodies. Molecular weight standards are indicated at the left. (D) Effect of cross-links of RR>KK mutated signal peptides with *p*Bpa exchanges; (strains and conditions in C) and D as in B)). Arrows indicate two TatA-cross-links with unaffected signal intensity. *, strong cross-links; *, weak cross-links; M, solubilized membrane fraction; E, elution fraction.

In an initial screen for Tat component interactions, we placed *p*Bpa at selected positions in the twin-arginine motif (A13, V14), the h-region (M26, F32), and the c-region (R36) of the signal peptide (Fig. 1A). HiPIP variants with *p*Bpa at these positions were still transported, as mature HiPIP was clearly detected in the periplasmic fractions of strains carrying the respective HiPIP variants (Fig. 1B). The V14*p*Bpa exchange clearly reduced translocation efficiency. The *in vivo* cross-link data with signal peptides that contained unaltered twin-arginines demonstrated that all three Tat components are in close proximity to the signal peptide (Fig. 1C). TatA and TatB showed very pronounced cross-links to positions of the h- and c-regions (M26, F32, R36), indicating close substrate contacts. TatC, which is well-known to recognize the twin-arginine motif [4,5], cross-linked to all positions with high efficiency, indicating that the complete signal peptide is in direct contact with TatC under active translocation conditions *in vivo*, which is in agreement with structural and functional characteristics of TatC [7,27,28,29].

We then used *in vivo* cross-linking to analyze the signal peptide interactions with RR>KK mutated Tat motifs (Fig. 1D). This exchange is known to lower dramatically the affinity to TatBC *in vitro* [4,29,30], resulting in a marked decrease or even block of translocation [26,31,32]. As expected, the KK-variants showed diminished overall cross-links to TatA, TatB or TatC at almost all positions, indicating that RR-motif-binding to the TatBC complex strongly enhances TatABC contacts to these positions. In control experiments, we ensured that RR and KK variants of HiPIP were all produced in comparable amounts (S1 Fig.). Residual contacts of KK-variants to Tat components are expected, as the RR>KK mutation does not fully abolish Tat transport in several test systems [6,33]. In line with this, we found that an optimization of the consensus hydrophobic residue by exchanges A13F as well as by A13*p*Bpa resulted in partial compensation of the translocational block caused by the initial RR>KK exchange (S2 Fig.). An aromatic residue at this position, which corresponds to the F in the consensus motif [1], has been previously shown to strongly enhance TatBC complex binding [5] and our data suggest that this enhanced binding is highly supportive for Tat transport. We were surprised by the clear TatA cross-links and by the fact that some TatA cross-links were not negatively affected by the RR>KK exchange (A13*p*Bpa and V14*p*Bpa; Fig. 1D). Together, these initial data raised the possibility for “basal” signal peptide TatA contacts throughout the signal peptide that are TatBC-independent, while only TatA interactions with the h- and c-regions of the signal peptide are intensified in a TatBC-dependent way that is indicative for later contacts.

**Fig 2:**
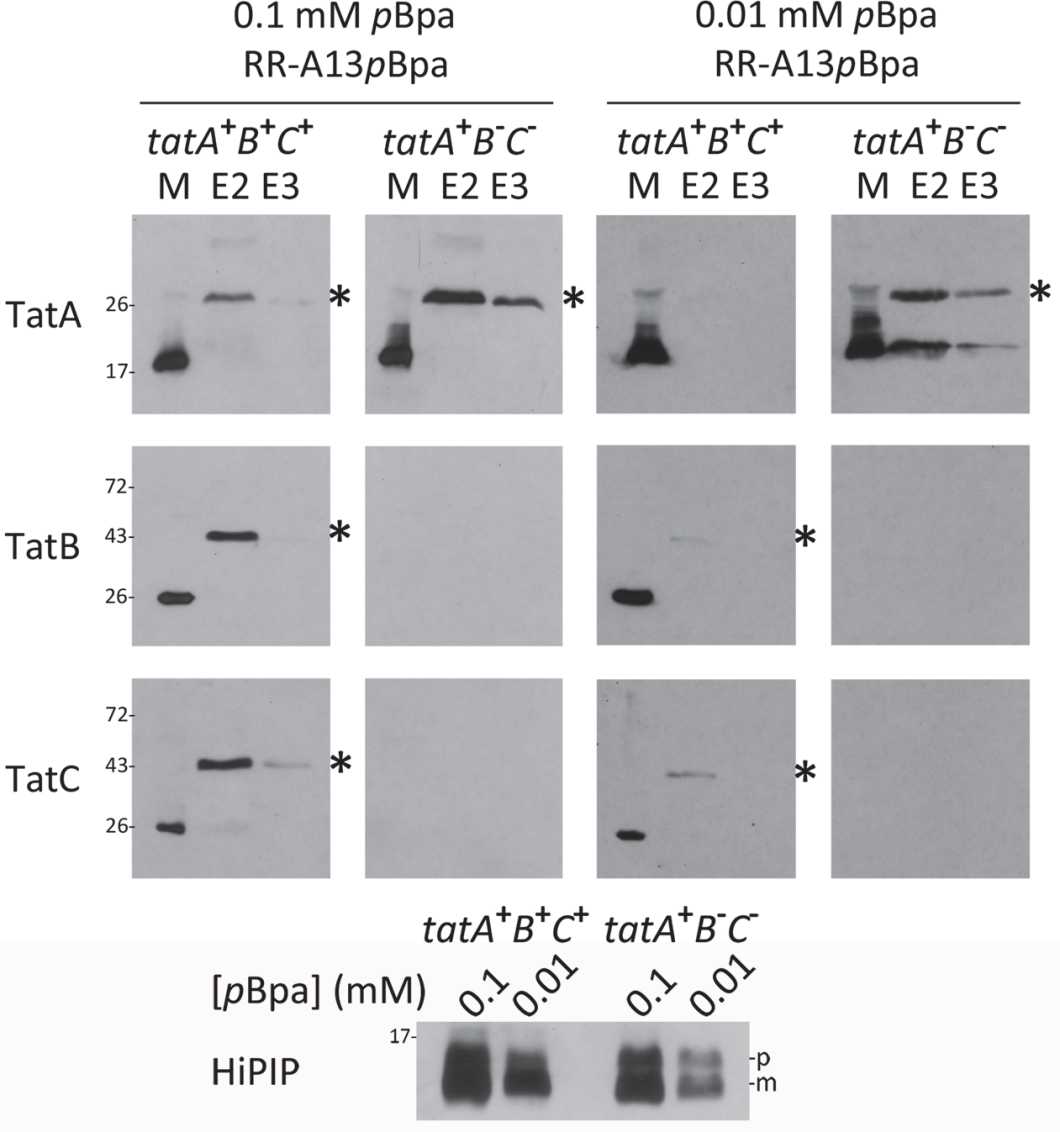
The TatA/Tat signal peptide contact does not require the TatBC components. Comparison of HiPIP-TatA cross-links in the presence/absence of TatBC using *p*Bpa at position A13. Two *p*Bpa concentrations (0.1 mM and 0.01 mM) in the growth medium were used to analyze effects of lowered HiPIP concentration. *tatA* ^+^ *B* ^+^ *C* ^+^: BW25113/pRK-*tatABC*/pEvol-pBpF /pEXH5*tac*-H6-A13*p*Bpa; *tatA* ^+^ *B* ^-^ *C* ^-^: JBdBC/pRK-*tatA*/pEvol-pBpF /pEXH5*tac*-H6-A13*p*Bpa. The bottom blot monitors the decrease of HiPIP levels by reduction of *p*Bpa concentration in the medium. HiPIP detection in crude extracts. Significant HiPIP degradation to mature size is due to unspecific proteolysis of the signal peptide. See Fig. 1 for further details.

We thus analyzed the contacts in more detail by directly comparing cross-links to TatA at position A13 in the presence and absence of TatBC (Fig. 2). We chose this position, as its contacts to TatA were not enhanced by the RR-motif and as—in contrast to V14*p*Bpa—the A13*p*Bpa variant was nicely translocated (Fig. 1). Again, we included detections of TatB and TatC cross-links in these analyses. Tat substrate cross-links to TatA clearly accumulated in the absence of TatB and TatC, which demonstrates that the TatA interaction can in principle take place prior to TatBC interactions, and that the substrate is not degraded when TatBC interactions cannot take place. When the Tat substrate level was reduced by lowering the *p*Bpa concentration, the TatA cross-links disappeared in the functional Tat background and still accumulated in the TatBC-deficient strain (Fig. 2, right panel). The decrease of substrate-level by lowering [*p*Bpa] is accompanied by increased translational stop, which at this position results in a hydrophilic 12-residues peptide that did not influence the analyses.

**Fig 3:**
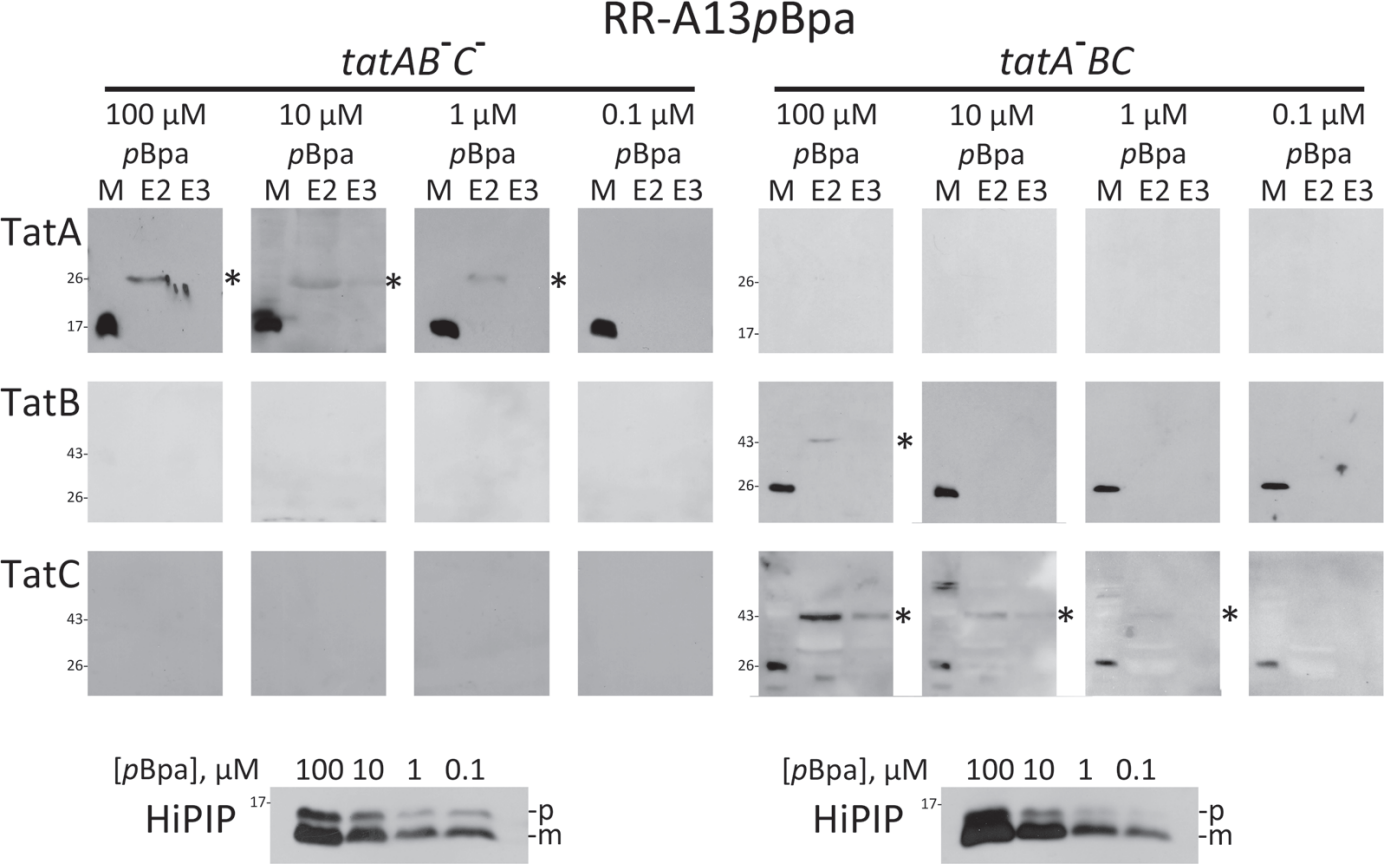
Substrate cross-links to TatA and TatC have comparable detection limits when substrate levels are gradually decreased. Detection of cross-links of RR-HiPIP with *p*Bpa at position A13 (RR-A13*p*Bpa) either to wild-type level non-recombinant TatA in the absence of TatBC (left section: *tatAB* ^-^ *C* ^-^, strain JBdBC) or to wild-type level non-recombinant TatBC in the absence of TatA (right section: *tatA* ^-^ *BC*; strain JARV16). Note that TatA and TatC cross-links are detectable at lowered substrate concentrations as achieved with 1 μM *p*Bpa in the medium. TatB cross-links are depleted below detectability already with 10 μM *p*Bpa, whereas TatA and TatC cross-links both become non-detectable with 0.1 μM *p*Bpa. Bottom blots: SDS-PAGE-Western blot detection of HiPIP in extracts of cells grown in the presence of indicated *p*Bpa concentrations. The bottom blots monitor the decrease of HiPIP levels by reduction of *p*Bpa concentration in the medium, as described in Fig. 2. p, precursor of HiPIP; m, mature form of HiPIP. See Fig. 1 for further details.

These data prompted an analysis of the lower limits of cross-link detectability, which showed that the observed TatA cross-links were also detectable with wild-type levels of the Tat components and very low substrate concentrations. Substrate cross-links in Tat-inactivated strains deficient in either TatA/E (strain JARV16) or TatB/C (strain JBdBC) were compared (Fig. 3). While the first strain monitors a TatA-independent TatBC interaction, the second strain monitors a TatBC-independent TatA interaction. Substrate levels were lowered by gradually lowering [*p*Bpa] until cross-links were not detectable anymore. The data clearly demonstrated that—at wild-type Tat component levels—the TatBC-independent TatA cross-links were detected with the lowest substrate levels that could be used to detect TatC-cross-links (1 μM *p*Bpa).

**Fig 4:**
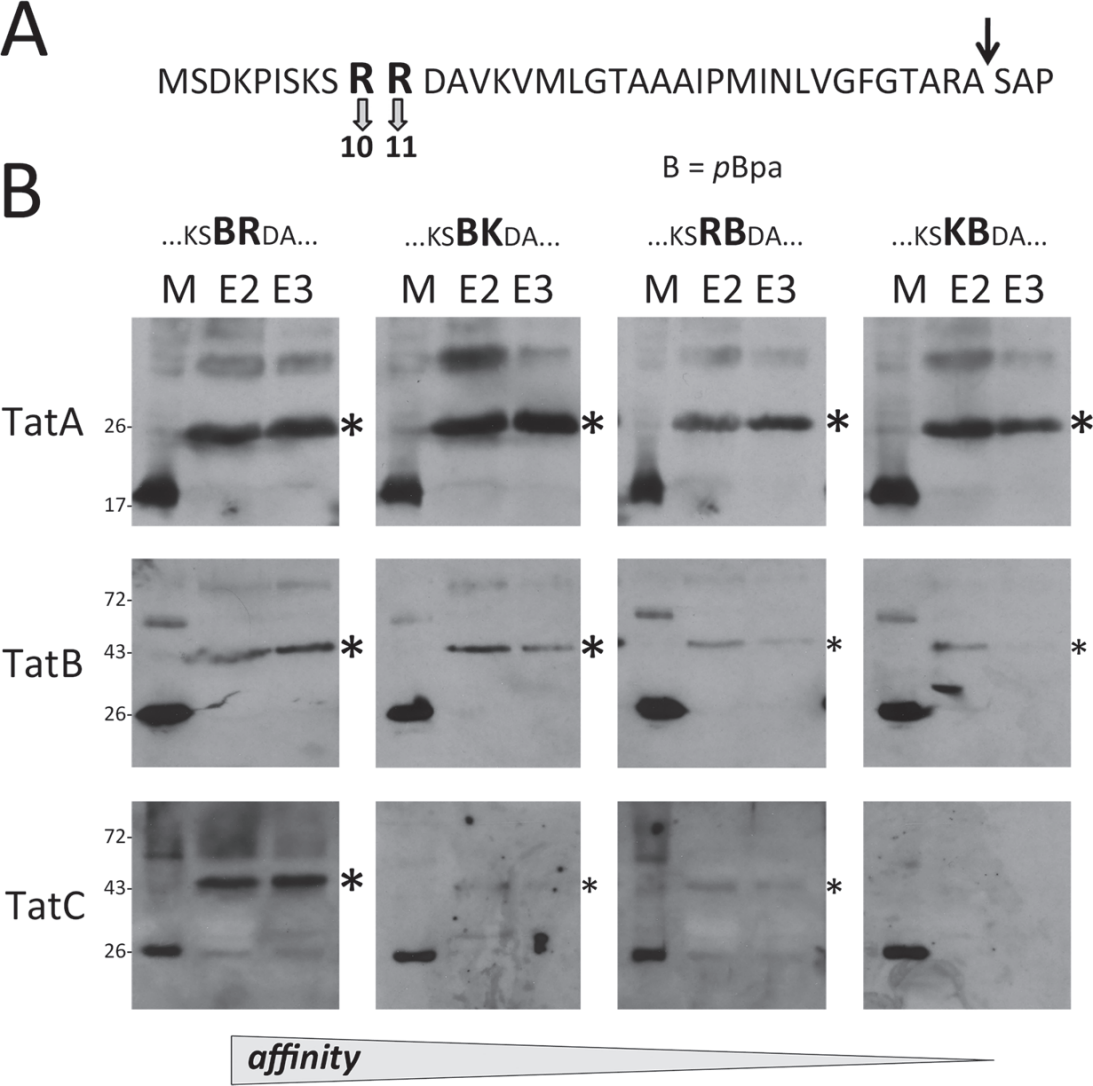
TatA does not recognize the RR motif. (A) Positions of arginines in the twin-arginine motif that were mutated for experiments shown under B). (B) Analyses of cross-links to Tat components with *p*Bpa (abbreviated “B”) at indicated twin-arginine motif positions (expression system and [*p*Bpa] as in Fig. 1). Additional R>K exchanges are indicated. A decrease of TatBC cross-link intensities relates to decreasing affinities as indicative for the twin-arginine motif recognition (indicated at the bottom) that is confined to TatBC.

As only TatBC-containing complexes are known to recognize twin-arginine motifs, we analyzed the relevance of the twin-arginines for the TatA cross-links with four mutated motifs (*p*Bpa abbreviated by B): BR, BK, RB, KB. Direct *p*Bpa substitutions of the conserved arginine residues of the RR motif had not been examined in previous *in vitro* cross-linking analyses and most likely were not generated because they were expected to abolish Tat interactions (albeit very sensitive translocation assays could demonstrate residual Tat transport even with RR>KK mutations; [6,33]). The results showed highly efficient TatA-cross-linking to *p*Bpa placed at both positions of the twin-arginines, whereas cross-links to TatB and TatC were gradually reduced in the order BR > BK > RB > KB, demonstrating that only TatBC components are involved in the recognition of the twin arginines (Fig. 4AB). Still remarkable TatBC cross-links in case of the BR motif reflect the high sensitivity of the cross-link detection. The KB-motif gave no detectable cross-links to TatC. The affinity-decrease in the order BR > BK > RB > KB highlights the preference of R over K as well as the outstanding importance of the positive charge at the second position for the motif recognition, which agrees with previous studies [6,31,33,34,35].

**Fig 5:**
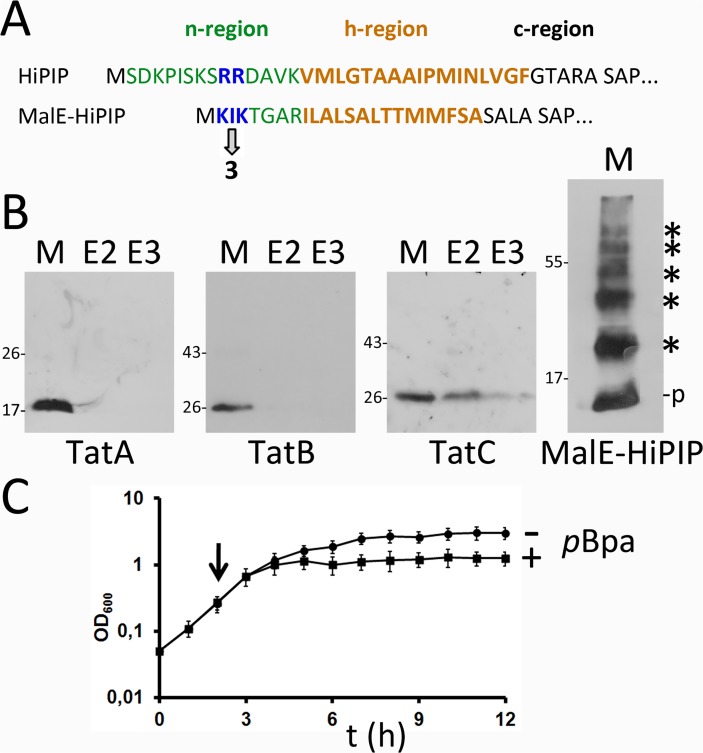
TatA does not cross-link to the signal peptide of the Sec substrate MalE. (A) Position of the *p*Bpa cross-linker in the MalE signal peptide relative to the position of the HiPIP signal peptide RR-motif. n-, h-, and c-regions are indicated (B) Cross-linking analysis of the MalE-signal peptide HiPIP fusion (expression systems and [*p*Bpa] as in Fig. 1). As control, the fusion was detected in membranes by HiPIP-specific antibodies. It formed multiple cross-links with its N-terminal *p*Bpa, indicating full-length of the signal peptide. See text for more details. (C) Growth defect upon production of MalE-HiPIP-I3*p*Bpa, pointing to jamming of the Sec translocon by Sec-targeted folded HiPIP. + *p*Bpa, Production of MalE-HiPIP-I3*p*Bpa was induced by addition of 0.1 mM *p*Bpa to the growth medium at the indicated time point. - *p*Bpa, negative control without *p*Bpa addition.

As the RR-motif was irrelevant for the TatA interaction, we assessed Tat specificity by replacing the Tat signal peptide of HiPIP by the Sec signal peptide of MalE (MalE_sp_-HiPIP; Fig. 5). A *p*Bpa was positioned in between the two K residues of the n-region of the MalE signal peptide (Fig. 5A), which with respect to the distance to the h-region corresponds to the above tested RR-positions in Tat signal peptides that clearly cross-linked with TatA (see direct comparison in Fig. 5A). The MalE_sp_-HiPIP precursor was stable *in vivo* and many cross-links could be generated that proved the reactivity of the *p*Bpa in the signal peptide, but no cross-link to TatA, suggesting that TatA differentiates between Tat and Sec signals by a distinct mechanism (Fig. 5B, right blot). Differences in other details could be involved, such as in the length of specific regions or in hydrophobicity, which are known to contribute to pathway preferences ([36,37,38]). An interesting side aspect is that MalE_sp_-HiPIP caused a severe growth inhibition, most likely due to jamming of the Sec translocon by the tightly folded HiPIP domain (Fig. 5C).

### Preparation of soluble TatA and demonstration of substrate-binding *in vivo* and *in vitro*

**Fig 6:**
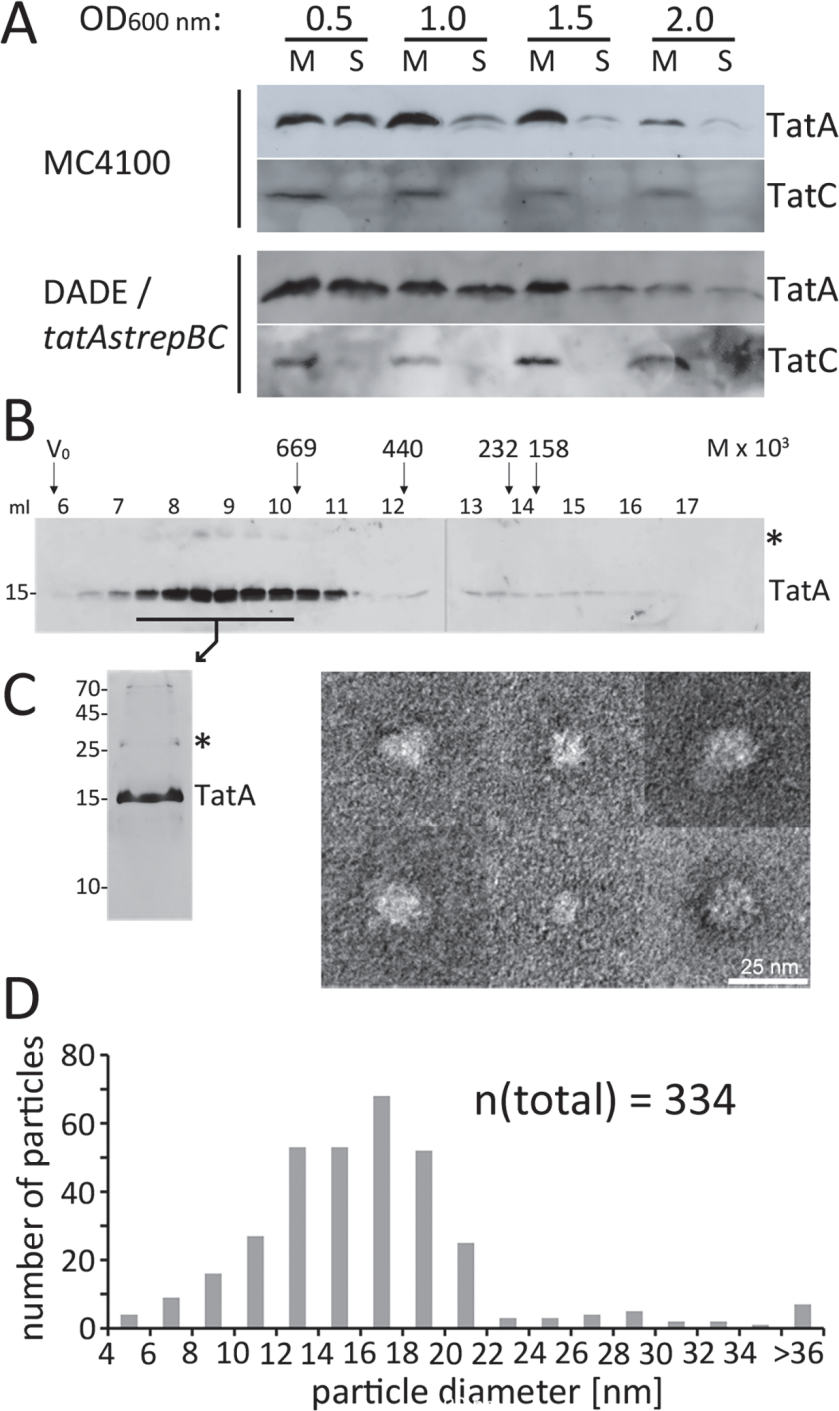
Membrane-detached soluble TatA forms large micellar associations. (A) Soluble TatA is abundant after cell disruption of bacteria at early exponential growth phase. Distribution of TatA in the membrane and cytoplasmic fraction in preparations from cultures of strains MC4100 and DADE *tatA-strep-tatBC*, harvested at indicated optical densities. Fractionation control was done by detection of the polytopic membrane protein TatC. Samples were normalized to the same amount of cells. (B) Size exclusion chromatography (SEC) of TatA-*strep* as purified from the cytoplasm after wild-type level production (DADE *tatA-strep-BC*) indicates formation of high molecular weight complexes. Western blot analysis of elution fractions; SEC molecular weight markers are indicated at the top (thyroglobulin, 669 kDa; ferritin, 440 kDa; catalase, 232 kDa; aldolase, 158 kDa). (C) Silver-stained SDS-PAGE of purified TatA-*strep* and analysis of the TatA particles by electron microscopy. Six typical TatA assemblies are shown; see supplement S4 Fig. for an overview micrograph. (D) Statistical analysis of TatA particle diameters based on 334 measured particles. M, membrane fraction; S, soluble fraction; *, TatA dimer. Molecular weight marker positions (in kDa) are indicated on the left of SDS-PAGE blots.

To address the TatBC-independent substrate/TatA interaction biochemically by a second, independent approach, we used detergent-free soluble TatA. Significant portions of TatA can be readily extracted from membranes without the use of detergents [39,40], which may be due to the very short membrane anchor or a population of soluble TatA that interacts with TatC at the membrane surface [21,41]. Indeed, soluble TatA has been observed in studies with archaea, bacteria, and plant plastids, strongly suggesting that TatA generally has the tendency to form soluble associations in biochemical preparations (*Streptomyces lividans*, *Bacillus subtilis*, *Haloferax volcanii*, as well as in the chloroplast stroma [23,39,42,43]). In *Streptomyces lividans* and in *Bacillus subtilis*, soluble TatA has been detected that had an intrinsic affinity to Tat substrates [22,23,41]. We also detected significant amounts of soluble TatA after cell disruptions, especially during early exponential growth (Fig. 6A). In the case of *E*. *coli*, this soluble TatA may result from a release from cell membranes during cell disruption, as the density of non-recombinant soluble TatA was experimentally determined to be ∼1.21 g/ml, which is higher than the density of cytoplasmic membranes (∼1.18 g/ml) and significantly lower than the density of pure protein (∼1.3 g/ml) (S3 Fig.). Like in the case of *Bacillus* or *Streptomyces* species, soluble TatA preparations from *E*. *coli* are large associations of many TatA protomers that broadly eluted from gel permeation chromatographies with a maximum size of about 800 kDa (Fig. 6B). Electron microscopy of purified *Strep*-tagged TatA revealed flattened, approx. round particles without characteristic features that would have been expected for a defined complex (Fig. 6C, see S4 Fig. for functionality confirmation of the construct and an overview micrograph). A statistical analysis of these TatA associations showed largely variable diameters with a most abundant size of ∼18 nm (Fig. 6D). Also few tube-like structures occurred. Together, the EM analyses suggested variable protein micelles rather than a defined complex. Protein micelles are expected to be formed by spontaneous interactions of the hydrophobic N-termini that must be shielded from the aqueous surrounding. Hydrophilic TatA regions in protein micelles are thus likely to be surface exposed just as in the case of TatA at the inner face of the cytoplasmic membrane.

**Fig 7:**
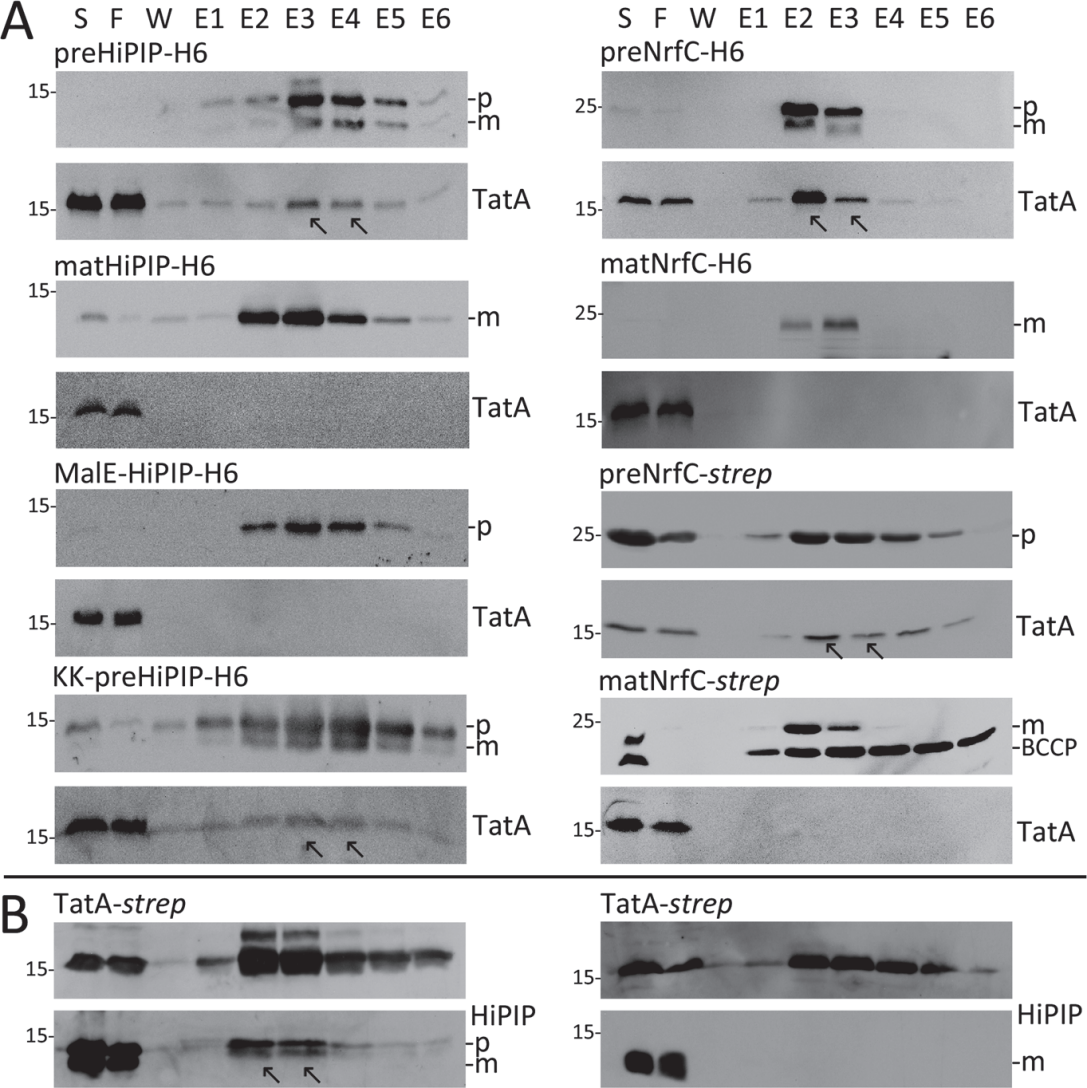
TatA/Tat substrate interactions in solution. (A) Soluble non-recombinant TatA co-purifies with the precursor of HiPIP and NrfC and not with their mature forms. Precursor and mature forms of HiPIP were produced in *E*. *coli* strain MC4100 using pEXH5*tac*-H6 and pEXH5*tac*-mat-H6, respectively. The MalE-signal peptide-HiPIP fusion protein was produced using pEXH5*tac*-*malE*(sp)-H6, and the RR>KK mutated HiPIP variant was produced using pEXH5*tac*-H6-KK. Precursor and mature forms of NrfC were produced using pBW-*nrfC*-H6, pBW-*nrfC*-mat-H6, pBW-*nrfC*-*strep* or pBW-*nrfC*-mat-*strep*, as indicated. Soluble protein (S), flow-through (F), wash (W) and elution fractions (E1-6) were analyzed by SDS-PAGE Western blotting, using antibodies directed against HiPIP, the H6-Tag (in case of NrfC), or TatA as indicated. (B) *In vitro* folded pure HiPIP precursor associates with tagged soluble TatA. 5 μM of precursor (left panel) or mature (right panel) HiPIP were added to crude soluble extracts containing wild-type level TatA-*strep* (from strain DADE *tatA-strep-tatBC*), incubated at room temperature for 20 min and used for affinity chromatography. Further analyses were carried out as in A), using antibodies directed against TatA or HiPIP, as indicated. Arrows in A) and B) indicate co-elutions. p, precursor; m, mature form; BCCP, biotin carboxyl carrier protein.

Irrespective the debatable origin of soluble TatA, for this project most important was that the preparations of soluble TatA were stable over weeks and thus allowed for the assessment of substrate interactions in the absence of detergents. We initially tested interactions with HiPIP. Like in the cross-linking experiments, we included the RR>KK variant of HiPIP to test whether the RR motif in the signal peptide contributes to a possible Tat specificity of binding. We also included a mature variant of HiPIP that lacks the signal peptide. All HiPIP variants were C-terminally tagged to allow affinity chromatography. Strain *E*. *coli* MC4100/pEXH5*tac*-H6 was used that contains non-recombinant natural TatA and hexahistidine-tagged HiPIP. Cells were disrupted, soluble fractions were prepared and HiPIP was affinity-enriched. Soluble TatA clearly co-eluted with HiPIP and was detected by an increase of the TatA signal in the fractions where HiPIP eluted. The interaction was stable enough to be detectable even with soluble TatA at wild-type level (Fig. 7A, left side). As expected, the co-elution depended on the signal peptide but not on the twin-arginine motif. Since it was possible that soluble TatA can in principle interact with any kind of signal peptide, we again included the Sec-signal peptide of MalE in our analyses as negative control. Like in the case of *in vivo* cross-linking, MalE_sp_-HiPIP did not show any co-elution with TatA, indicating that indeed the Sec-signal peptide does not support the HiPIP interaction with TatA.

HiPIP is a heterologous Tat substrate, and the observed interaction was therefore likely reflecting a general property of TatA that should be observable also with *E*. *coli* Tat substrates. To test this, we examined the interaction with the *E*. *coli* Tat substrate NrfC [44]. Strikingly, non-recombinant TatA was even more pronounced co-eluting with NrfC and the interaction clearly depended on the signal peptide (Fig. 7A, right side).

While the above interactions have been established *in vivo*, it was unclear whether TatA / Tat substrate interactions can also be generated *in vitro*, i.e. outside the living cell. To test this, 5 μM purified *in vitro* folded HiPIP were added to cytoplasmic fractions containing *Strep*-tagged TatA at natural levels (from a strain with a single-copy chromosomally integrated *tatA*-*strep*-BC operon). HiPIP clearly co-eluted with soluble TatA-*strep* in *Strep*-tactin affinity chromatography in a signal sequence-dependent manner (Fig. 7B). TatA thus can recognize folded HiPIP precursor *in vivo* as well as *in vitro*.

### The TatA/Tat substrate interaction involves determinants of the folded substrate domain and is important for efficient Tat transport *in vivo*

**Fig 8:**
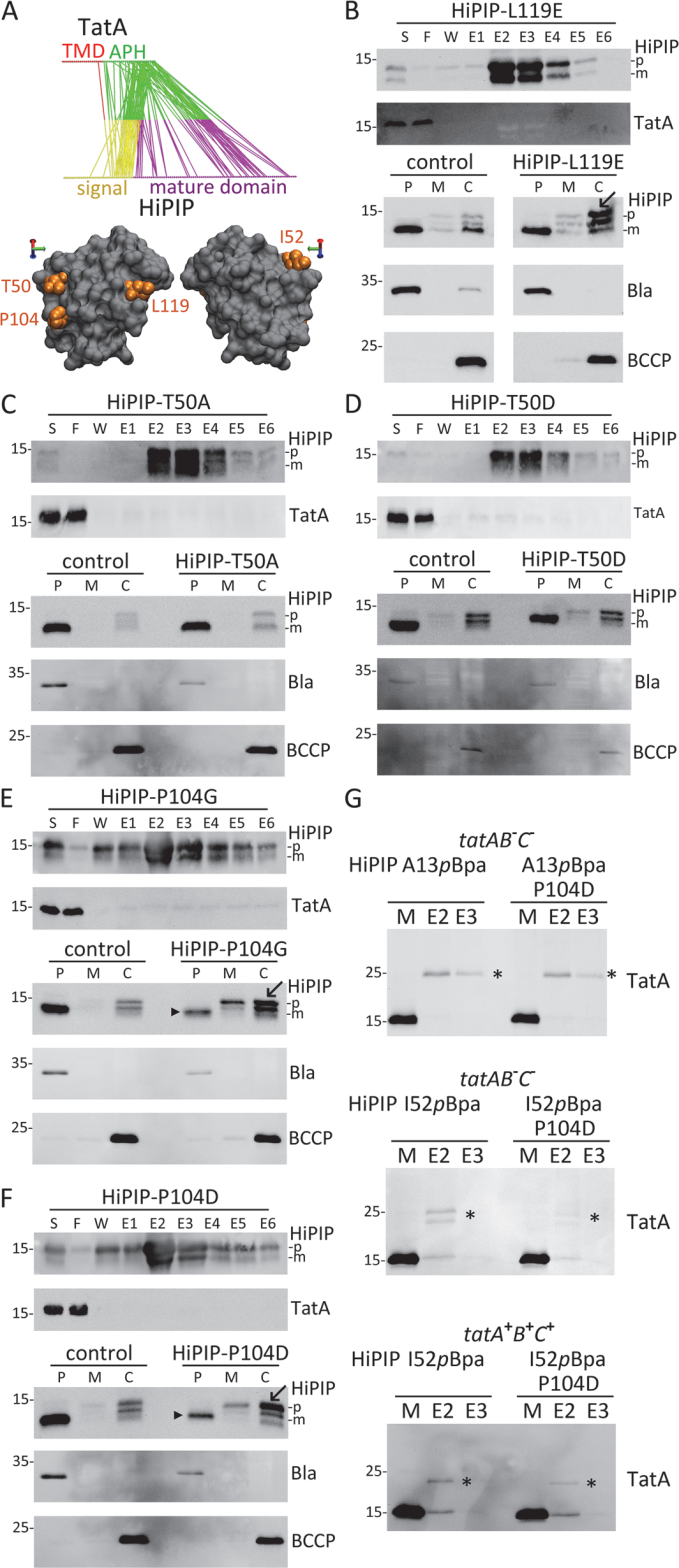
Identification of TatA-binding determinants on the surface of folded HiPIP. (A) Most frequent interactions observed in 50 independent molecular dynamics (MD) simulations of TatA-HiPIP interactions (upper diagram). Color code: yellow: HiPIP signal peptide; magenta: HiPIP mature domain; red: TatA trans-membrane domain; green: TatA amphipathic helix. The three residues on the HiPIP surface with most contacts are positioned on one side of HiPIP and the *p*Bpa exchange position I52 are highlighted (lower diagram). See S5 Fig. for a snapshot of a molecular dynamics simulation of TatA-HiPIP interaction. (B) – (F) Analysis of affinity purification, TatA co-elution (see Fig. 7 for experimental details) and *in vivo* translocation of HiPIP-L119E (B), -T50A (C), -T50D (D), -P104G (E), and -P104D (F). *In vivo* translocation was examined as described in Fig. 1B. For all subcellular fractionations, the periplasm control (β-lactamase, Bla) and the cytoplasm control (BCCP) are shown. Arrowheads indicate strongly reduced translocation and arrows indicate accumulating precursor HiPIP. p, precursor; m, mature; P, periplasm; M, membrane; C, cytoplasm; markers are indicated on the left of the blots. (G) Cross-linking of position I52 of the HiPIP mature domain to TatA is affected by the HiPIP-P104D mutation, whereas the interaction with the signal peptide A13 position remains unaffected. Analysis of HiPIP cross-links to wild-type level TatA in the absence of *tatBC* (*tatAB* ^−^ *C* ^−^, strain: JBdBC) or with slightly increased levels of TatABC (pRK-*tatABC*), (*tatA* ^+^ *B* ^+^ *C* ^+^, strain: MC4100). *p*Bpa was positioned in the signal peptide (A13, upper blot) or in the mature domain (I52, lower two blots), and the effect of the TatA interaction-weakening HiPIP-P104D exchange on cross-link intensities was analyzed in parallel. *, cross-links.

To address the physiological function of an alternative TatA-dependent targeting of substrates to the translocon, we searched for mutations that specifically affected TatBC-independent TatA/Tat substrate interactions to examine possible effects on transport. Candidate residues with possible importance for TatA/Tat substrate interactions were identified first by molecular dynamics (MD) simulations of HiPIP/TatA interactions in the membrane (Fig. 8A). The simulations predicted that the hydrophobic part of the HiPIP signal peptide can dip quite deeply into the membrane bilayer with its h-region kinked at a proline residue. Statistical analyses revealed possible TatA contact sites in the C-terminal half of the signal peptide and on the mature domain of HiPIP. As all Tat components contact the signal peptide (Fig. 1), and as TatBC complexes are not known to specifically recognize epitopes of mature domains and rather bind the RR-motif, the TatA contacts to the mature HiPIP domain raised the possibility that the mutation of mature domain surface residues could selectively weaken the TatBC-independent TatA recognition. The predicted contacts between TatA and the mature domain of HiPIP were all positioned on the same surface side of this small protein (Fig. 8A). Among the most prominent contacts in 50 independent simulations were contacts to T50, P104, and L119. To examine whether these contacts contributed to the affinity of TatA to HiPIP, we tested the system with T50D, T50A, P104D, P104G, and L119E exchanges (Fig. 8B-F). All mutations diminished the interactions to soluble TatA to an extent that compromised the co-elution. Co-purification requires strong interactions and a slow dissociation, and therefore an abolished co-elution can result already from minor changes that may not necessarily result in physiological effects *in vivo*. Nevertheless, the tested HiPIP mutations were promising candidates for mutations that might sufficiently affect the TatBC-independent TatA interaction to disclose a physiological role of the interaction. We therefore analyzed effects of these mutations on translocation efficiency. In control experiments, we ensured that the surface mutations did not compromise folding of HiPIP (S6 Fig.). While the T50A and T50D mutations did not affect transport, the other three mutations clearly caused an accumulation of precursor in the cytoplasm, and in the cases of P104D and P104G even in the membrane. With P104D of P104G mutations, the accumulation of precursor was clearly attributable to a lowered translocation efficiency, as the signal of transported mature HiPIP in the periplasmic fraction was strongly reduced (Fig. 8EF). To finally examine whether transport-compromising exchanges on the HiPIP surface did indeed reduce the TatA contacts *in vivo*, we carried out an *in vivo* cross-link experiment and monitored the TatA interactions with the signal peptide and the mature domain of HiPIP with or without the P104D exchange. We placed *p*Bpa either at position A13 of the signal peptide or at position I52 of the mature domain, which was recently shown by us to contact TatA and TatB *in vivo* [45]. While the signal peptide interaction with TatA was unaffected by the P104D exchange, the I52*p*Bpa cross-link to TatA was clearly reduced, and this reduction was independent of TatBC (Fig. 8G). The residual cross-link indicates that the exchange does not completely abolish the interaction, which is expected for contacts that are mediated by multiple interactions.

## Discussion

### *In vivo* cross-linking data indicate a TatBC-independent TatA interaction

At the beginning of this study we used *in vivo* photo-activatable site directed cross-linking tools for the analysis of the protein translocating Tat system. So far, such Tat system analyses have been only carried out *in vitro* [4,5,20,46,47]. The *in vivo* system included an affinity chromatography-based enrichment of cross-links that increased the signal intensities of the Western-blot based assay to levels comparable to *in vitro* assays that can use additions of radiolabeled substrates [4]. The *in vivo* assay permitted the detection of weak cross-links of RR>KK mutated signal peptides to TatB and TatC (Fig. 1)—something that could not be detected so far *in vitro*, albeit a residual translocation activity with RR>KK mutated signal peptides has been shown *in vivo* by highly sensitive transport assays [6,33], and we similarly observed with HiPIP that RR>KK mutated signal peptides can mediate residual transport if only the Tat motif is optimized by a A13F or A13*p*Bpa mutation (S2 Fig.). The *in vivo* cross-links with functional Tat substrates in the presence of active TatABC components monitor any interactions of the complete translocation path and it was thus possible to detect TatC cross-links to positions in the h- and c-region of the twin-arginine signal peptide (Fig. 1), which agrees with a deep contact of the signal peptide with TatC as supported by the TatC structure, protease protection studies, and the signal peptide insertase activity of TatC [27,28,29]. This agrees also with a recent report of a PMF-dependent TatC cross-link of a F32*p*Bpa exchange in the HiPIP signal peptide [45].

Cross-linking analysis with *p*Bpa at RR or KK positions showed that TatC can distinguish arginines from lysines, with the second arginine position being much more important than the first, with a tolerance for even uncharged substitutions at the first position (Fig. 4). This fits to the analysis of Tat transport with a highly sensitive reporter system that could detect the same preferences [33]. In contrast to TatC, TatA did not show any recognition of the arginines and cross-linked to all *p*Bpa substitutions at these positions with similar efficiencies. The interaction was nevertheless completely abolished when the Tat signal peptide was exchanged by the Sec signal peptide of MalE, suggesting a role of known Tat determinants other than the twin-arginine motif ([38] and Fig. 5). However, a strict differentiation between Sec and Tat signal peptides might even not be necessary *in vivo*: A targeting to the Sec translocon can occur either via SRP or SecA-mediated pathways, and for both pathways a signal peptide binding can already take place at the ribosome, which would prevent any TatA interaction [48,49,50]. TatB cross-links gave some intermediate result, which may be explained by the reported formation of TatAB complexes *in vivo* [19,51,52], that add to the well-known TatBC complexes [53].

Importantly, TatA cross-linked to Tat substrates independently of TatBC and at lowest substrate concentrations that were required to see TatC cross-links at wild-type Tat levels, which was suggestive for a delivery function of substrate-associated TatA *in vivo* (Figs. 2 and 3). This corresponds to the assembly of complete TatABC-Tat substrate translocons, as it is known that TatBC/Tat substrate recognition induces a recruitment and clustering of TatA at the translocon [10]. The resulting substrate-containing TatABC complex has been recently detected in *E*. *coli* by BN-PAGE analyses at ∼600 kDa [9]. The recognition of the RR motif by TatBC complexes is well-documented [4,5,6,7] and in full agreement with our cross-linking results (Fig. 1 and 4).

### TatA binds the signal peptide as well as the mature domain of certain Tat substrates

**Fig 9:**
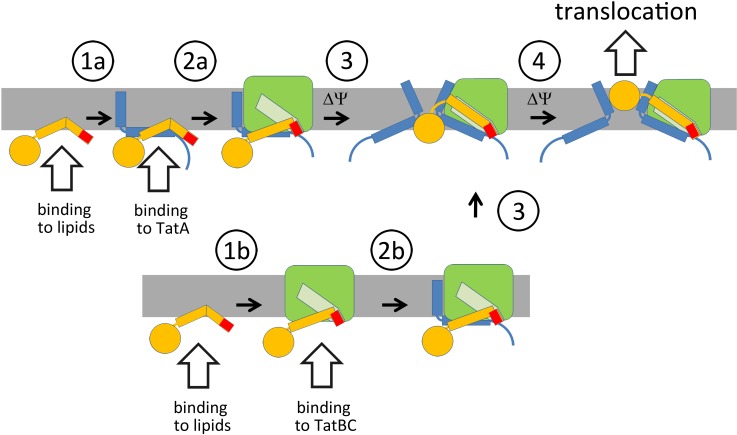
Interactions during Tat translocon assembly. Tat substrate signal peptides such as from HiPIP or NrfC can spontaneously interact with membrane surfaces where they can either encounter free TatA (1a) or TatABC complexes (1b). TatA-bound substrates expose the RR-motif that is recognized by TatBC in the TatABC complex (2a). More TatA is recruited, possibly when TatBC imposes a force to the Tat substrate, which could influence TatA orientations and the local membrane curvature (3). Such a force could result from the known binding of larger signal peptide regions into the membrane-dunking binding-site regions at TatBC. Sufficient TatA recruitment finally permits translocation (4). If Tat substrates are first bound to TatBC, either after membrane interaction or directly (1b), the TatA assembly has to take place thereafter (2b). Such a binding may be more relevant for Tat substrates that do not readily interact with membranes, as the likelihood to first encounter TatA in membranes is high. “ΔΨ” designates steps that likely require the membrane potential. Color-code: Tat substrate, yellow; twin-arginine motif, red; TatA, blue; TatBC, green; binding-site for the signal peptide in TatBC, light green.

As the cross-linking data can only demonstrate a close proximity between components, it was important to show that this corresponded to a binding of the proteins and that HiPIP is representative for other natural Tat substrates as well. This was achieved by co-purification and by including a second natural Tat substrate, *E*. *coli* NrfC. We found that soluble TatA is an ideal tool for studying the interactions: it is stable in solution, its purification does not require the use of detergents that could abolish interactions, and – most importantly – it binds Tat substrates (Figs. 6 and 7). With HiPIP as well as with NrfC, binding depended on the signal peptide. Since HiPIP is the structurally much better characterized Tat substrate, we used it to analyze the binding in more detail and we could show that a RR>KK exchange still allowed for an interaction whereas a MalE Sec signal peptide exchange diminished the affinity of HiPIP to TatA. These data agree with results reported in studies on the *Streptomyces lividans* Tat system [21]: In that study, recombinantly overproduced TatA from the soluble fraction bound Tat substrates with high affinity whereas a Sec signal peptide lowered the affinity by several orders of magnitude. The analysis of RR>KK exchanges was not reported in that study, which measured the affinities using surface plasmon resonance biosensors. A second report on a direct TatA/Tat substrate interaction comes from studies with *Bacillus subtilis*, where the Tat substrate PhoD interacts with a PhoD-specific TatA_d_ [41]. In this case, positively charged residues in the RR-motif contributed to the affinity without being essential for the TatA interaction. This motif has the sequence DRRKFIQ in PhoD, and R>K exchanges had no effect and even a RRK>AAA exchange retained significant TatA affinity. These results are in full agreement with our findings and raised the question, what accounts for the specificity in the PhoD-TatA_d_ interaction. Unexpectedly, in our experiments we found that mature domain can be highly important for the interaction. As the HiPIP and TatA structures are known, we could carry out MD simulations that identified three HiPIP surface residues that frequently contacted TatA, and single amino acid exchanges in these positions disabled the co-elution of TatA in affinity chromatographies. Such an effect on co-elution could already be due to a minor reduction in affinity, without abolishing a functional interaction *in vivo*. It was therefore important to assess, whether the mutations also affected Tat transport *in vivo*. Indeed, translocation assays showed that two of the three positions clearly affected translocation efficiency, strongly suggesting that the TatBC-independent TatA interaction is relevant for Tat transport (Fig. 8). With the P104D exchange, which affected transport most strongly, we showed that indeed the abolished TatA co-elution correlated with reduced TatA cross-links to the surface of the HiPIP mature domain (Fig. 8G). This was observed in the absence as well as in the presence of TatBC, confirming the TatBC-independence of this interaction. Interestingly, the signal peptide interaction with TatA was not affected by the exchange at the mature domain surface, suggesting that the two regions are both recognized by TatA, and only if both are bound, the interaction is strong enough to permit a co-purification. This identification of surface residues with importance of TatBC-independent TatA interactions and Tat transport indicates a physiological contribution to the transport process and it shows that it is most likely the interplay of signal peptide and mature domain characteristics that promotes TatA/Tat substrate interactions and thereby confers Tat substrate specificity. We believe that this is the key to understand the substrate-specific role of several TatA components, such as NosZ-transport-specific TatE in *Pseudomonas stutzeri* or PhoD transport-specific TatA_d_ in *Bacillus subtilis* [23,54]. Future studies will have to focus on the exact determinants for mature domain interactions which are likely to play important roles for the recognition of specific Tat substrates, such as HiPIP, NrfC, PhoD or NosZ. The delivery of Tat substrates by TatA to the Tat translocon may explain the very distinct distribution of TatA and functional TatABC translocons. TatA associations are found distributed in the cytoplasmic membrane without a clear preference to specific subcellular regions [16,19,55]. In contrast, the RR-dependently formed TatC-containing translocation sites are located in few, often polar or sub-polar small foci [17,19]. TatA clearly is part of the active translocons, but the TatA/Tat substrate interaction and the improvement of transport suggest that the freely-diffusing population of TatA can contribute to targeting as outlined in Fig. 9, which shows our current working model for the sequence of events during Tat transport. This model includes the accepted current model and only extends it by the herein described TatA interaction. Tat substrates will first interact with membranes [56,57,58,59,60] and in *E*. *coli*, the first Tat component encountered by membrane-interacting Tat substrates is likely to be TatA. TatA exists in 50-fold molar excess [61] and the freely diffusing TatA is known to form tetramers, whereas TatC forms higher oligomers, further reducing the occurrence to a few spots in the cell [17,19]. Accordingly, TatA interactions are detectable already at very low substrate levels, at the same concentrations that are required to detect TatC interactions (Fig. 3). Early TatA interactions certainly do not substitute the later RR-motif recognition by TatC and are not expected to be essential for Tat transport, but nevertheless these interactions can positively affect Tat transport (Fig. 8). They may thus facilitate translocation by promoting TatBC interactions, accelerating the later TatA-recruitment step, and/or by protecting membrane-interacting signal peptides. Future studies will have to uncover the mode by which these early TatA/Tat substrate interactions contribute to Tat transport.

## Materials and Methods

### Strains and growth conditions

*Escherichia coli* strains MC4100 ara^R^ [16], its derivatives, BW25113 [62], and JBdBC (this study) were used for *in vivo* cross-linking and physiological studies. *E*. *coli* XL1-Blue Mrf’ Kan (Stratagene) and BW23473 [63] were used for cloning. The bacteria were grown aerobically at 37°C on LB-medium (1% (w/v) tryptone, 1% (w/v) NaCl, 0.5% (w/v) yeast extract) in the presence of the appropriate antibiotics (100 μg/ml ampicillin, 25 μg/ml chloramphenicol, 15 μg/ml Kanamycin 12.5 μg/ml tetracycline).

### Plasmids and genetic methods

The single copy integration vector pAH120-P_*tat*_-*tatA-strep-tatBC* was generated by amplification of the tat genes (from the 3’ region of *tatA* to *tatC*) using the primers *tatA*-Bam-s-ATG-B-F (5′-AGG TGG GAT CCT GGA GCC ACC CGC AGT TCG AAA AAT AAG CAG GTG TAA TCC ATG TTT GAT ATC GGT TTT AGC GAA C-3′) and pABS-*tatC*-BglII-R (5′-ATA TAG CGC GCT TAT TCT TCA GTT TTT TCG CTT TC-3′), followed by restriction with BamHI/BglII and ligation into the corresponding sites of a pAH120-P_*tat*_-*tatA-strep* derivative [40] in which a second BamHI site downstream of *tatA-strep* had been removed by QuikChange (Stratagene) PCR. pRK-*hip* was used for constitutive low-level expression of the *hip* gene from its own promoter [26]. pEvol-*p*BpF (courteously provided by Peter G. Schultz) was used to incorporate *p*Bpa at UAG stop codons [24,25,64]. When indicated, the plasmid pRK-*tatABC* [26] or its *tatBC*-deleted derivative pRK-*tatA* were used for constitutive moderate (15-fold) overproduction of TatABC or TatA, respectively. HiPIP was produced constitutively by plasmids pEXH5*tac* or pEXH5*tac*-KK, respectively [65], in which a C-terminal His_6_-tag-encoding sequence was fused to the *hip* gene by cloning the corresponding PvuI fragment from pEXH7 into the PvuI-digested pEXH5*tac* and pEXH5*tac*-KK [66], resulting in the vectors pEXH5*tac*-H6 and pEXH5*tac*-KK-H6, respectively. To construct HiPIP variants with amino acid exchanges, desired codons were introduced at specific sites by QuikChange mutagenesis (primers listed in S1 Table). The MalE-signal sequence-HiPIP-fusion constructs were created with and without the *amber* stop codon at position I3 of the MalE signal peptide (S2 Table). For construction of pEXH5*tac*-mat-H6, the mature domain-encoding sequence was amplified with pEXH5*tac*-H6 as template and used to substitute the precursor-encoding sequence in the template vector (see S2 Table). The L119E mutation was generated by amplification of the *hip* gene with mutational primers (see S2 Table). The pBW22 expression system was used for rhamnose-inducible expression of *nrfC*-H6 or *nrfC*-*strep* [67].

The strain JBdBC has been generated by deleting the *tatBC* genes in BW25113 by the method of Datsenko and Wanner [62].

To construct strain DADE *tatA-strep-tatBC*, the *tatA-strep-tatBC* operon, expressed under control of the *tatA* promoter (pAH120-P_*tat*_-*tatA-strep-tatBC*) was single copy integrated into the λ_*att*_-site of the *tat* deletion strain DADE [68], using the method of Haldiman and Wanner [63]. All constructs were confirmed by restriction analyses and sequencing.

### Biochemical methods

For the site-specific *in vivo* incorporation of *p*Bpa at the position of the amber stop codon by the pEvol System, *p*Bpa (Bachem, dissolved in 0.5 M NaOH) was added with indicated concentrations simultaneously with 100 μM arabinose three hours prior to UV-cross-linking and harvest of the cells. For comparative analysis of HiPIP production 6 ml of the respective cell suspension was removed before cross-linking procedure. Cells were suspended in 1 ml Tris HCl, pH 8.0, disrupted by sonication and the supernatant was used for SDS-PAGE after sedimentation of cell debris. Cross-linking of *p*Bpa was induced by irradiation of the cultures with UV light at 365 nm wavelength for 30 min at room temperature. Membranes were prepared from harvested cells and solubilised in buffer containing 3% SDS for 30 min and an adjacent dilution to 0.1% before affinity chromatography. Non-solubilised material was separated by ultracentrifugation (30 min, 130,000 x g, 4°C). Purification of His_6_-tagged proteins under denaturing conditions was carried out by Ni-NTA metal affinity chromatography as described elsewhere [69]. All purification buffers contained 0.1% SDS. Wash- and Elution-fractions were 10-fold concentrated by trichloroacetic acid precipitation and the presence of Tat-components and their cross-links was assessed by Western blotting as described below. Co-elution experiments were achieved by Ni^2+^-affinity chromatography [69] or *Strep*-Tactin Superflow chromatography (IBA).

The distribution of soluble and membrane-bound TatA at wild-type level was analyzed with cells harvested at different time points during growth (OD_600nm_ = 0.5, 1.0, 1.5, 2.0) in cell-density normalized amounts. Cells were resuspended in 20 mM Tris HCl, pH 8.0 and disrupted by sonication. After removal of cell debris (15,000 x g, 10 min, 4°C), membranes were sedimented (130,000 x g, 30 min, 4°C) in 200 μl fractions and resuspended in the same amount of buffer.

Purification of soluble TatA was performed with cells harvested in the early exponential growth phase (OD_600nm_ = 0.5). Cells were resuspended in 10 mM Hepes (4 ml/g cells), pH 8.0 and 150 mM NaCl, and passed twice through a French Press cell at 138 MPa. Cell debris and membranes were removed by centrifugations (20,000 x g, 15 min, 4°C and two times 130,000 x g, 1 h, 4°C). TatA-*strep* from the soluble fraction was purified by affinity chromatography on *Strep*-Tactin Superflow (IBA) columns according to the supplier′s protocol, but using the above mentioned buffer. Elution fractions containing TatA-*strep* were pooled, concentrated and further purified by size exclusion chromatography using a Superose 6 column. Inclusion bodies of precursor HiPIP were produced and folded as described previously. Mature HiPIP was obtained from folded precursor HiPIP by thermolysin treatment (200 μg/ml) for 30 min on ice [26].

SDS-PAGE was carried out by the method of Laemmli [70]. Subcellular fractionations were obtained by an optimized osmotic shock procedure. Briefly, 50 ml exponentially growing cultures were sedimented (4,500 x g, 4°C), cells were resuspended in 20 ml 20% sucrose/10 mM Tris HCl, pH 8.0/1 mM EDTA, incubated for 10 min at room temperature, and again sedimented (4,500 x g, 4°C). The supernatant-free cell pellet was resuspended in ice-cold 1 ml 5 mM MgSO_4_ and incubated for 20 min on ice. Shocked cells were sedimented (9,500 x g, 4°C) and the periplasm (supernatant) was carefully collected. The pellet was resuspended and cytoplasm and membranes were further separated by disintegration and centrifugation steps as described previously [26]. Western blotting was arranged as described previously using polyclonal rabbit serum against purified HiPIP, against synthetic TatA, TatB or TatC peptides (C-terminal 17, 17, 16 residues, respectively), against β-Lactamase (Acris), YidC (donated by Andreas Kuhn, Hohenheim) and His-tags (Qiagen). The biotin carboxyl carrier protein was detected by a *Strep*-Tactin-HRP-conjugate (IBA). Cells were visualized by differential interference contrast (DIC) as described previously [16].

### Isopycnic ultracentrifugation

The different migration behavior of membrane-integral and membrane-free TatA was analyzed in a cesium chloride density gradient. 24.5 ml of a 2 M CsCl solution was over-layered with 0.5 ml of a membrane or soluble fraction derived from MC4100 culture grown to OD of 0.5. The samples were centrifuged at 360,000 x g for 20 h afterwards separated in 1 ml fractions and refractive indices measurements were achieved with a refractometer (RM40, Mettler Toledo). Densities were calculated as described [71].

### Electron microscopy

Purified cytoplasmic TatA preparations were used for electron microscopical analyses. TatA particles were adsorbed to carbon foil and negatively stained with 1% uranyl acetate, as is described elsewhere [72]. Microscope settings were applied as described previously [73].

### Molecular dynamics simulations

The molecular dynamics simulations were performed with the GROMACS simulation package [74], version 4.5.5. We used the MARTINI coarse-grained model [75,76] to simulate the lipids, amino acids and solvent. In all simulations, the system was coupled to a constant temperature bath [77] with a relaxation time of 1.0 ps. We performed our simulations at a temperature of 310 K. Periodic boundary conditions where applied to simulate bulk behavior. The time step used in the simulation was 20 fs. The dielectric constant in the simulations was ε_r_ = 15. The neighbor-list was updated every 10 simulation steps. The pressure was weakly coupled [77] to 1 bar with a relaxation time of 0.5 ps.

TatA was modeled using the MARTINI model for proteins, which qualitatively captures the chemical nature of each individual amino acid and implicitly includes the secondary structure. The secondary structure was modeled by both restraining proper dihedrals between four neighboring backbone beads with an harmonic potential and by altering the non-bonded interactions according to the imposed secondary structure (free in solution, or in a coil or bend the backbone has a more polar character than in a helix or -strand). Further details concerning this methodology can be found in the original publication [76]. In these simulations the secondary structure of TatA was obtained from a recently obtained NMR-structure [15] and translated according to the DSSP definition. The secondary structure of the HIPIP signal peptide was based on local hydropathy (http://gcat.davidson.edu/DGPB/kd/kyte-doolittle.htm) and the prediction that the signal peptide may have a trans-membrane helical region between LEU17 to ALA36 (http://www.cbs.dtu.dk/services/TMHMM). In here, we allowed additional flexibility of PRO17 by modeling PRO17 as a coil, and ILE23 and MET25 as a band. Residues SER1 to MET16 were modeled as a random coil. This structure of the signal peptide should be regarded as a first approximation. The signal peptide was fused with the X-ray structure of the mature domain (PDB 1hip) of HIPIP. For sake of optical convenience, the 4 resolved Fe-S complexes were modeled by 4 polar beads (P4) and kept into the binding pocket by a harmonic elastic network (force constant of 500 kJ nm^−1^mol^−1^) formed with all nearby residues (< 0.8 nm cutoff). To additionally conserve the structure/secondary structure of the mature domain an additional elastic network was constructed between the backbone beads of residues ALA40-GLY121 (0.5 > cutoff < 0.8 nm).

A 6x6 nm piece of the bacterial membrane was modeled by symmetrically placing mimics of DPPE (102), DPPG(26) and Cardiolipin (8) in a bilayer configuration. The model of cardiolipin is described elsewhere [78]. Sodium counter-ions were added to neutralize the system.

The clustering between TatA and HIPIP was studied by performing 50 independent simulations of up to 4 microseconds [75] starting from different random initial velocities. An interaction between HIPIP and TatA is defined by a distance less than 0.5 nm between two atoms. For each simulation we listed the 3 most frequent observed interactions.

## Supporting Information

S1 Fig Control blots for site-directed UV-activated *in vivo* cross-linking. (A) During affinity chromatography, specific cross-links elute in fractions E2 and E3. Shown is the immunoblot-detection of cross-links of RR-HiPIP with a M26*p*Bpa substitution to TatB, using antibodies recognizing TatB. M: solubilized membranes, W: last wash fraction, E1-E4: elution fractions. (B) Without UV irradiation or *p*Bpa, no shifted bands can be detected in the elution fractions. For this important control, we chose HiPIP M26*p*Bpa, which gives strong cross-links to all Tat components after UV irradiation (see Fig. 1A). No cross-links could be detected without UV irradiation or *p*Bpa (+ *p*Bpa/-UV, −*p*Bpa/-UV, left blots). We also analyzed the UV-dependence of the cross-links for the HiPIP A13*p*Bpa variant that was the most important variant in our studies. As expected for the UV-activatable cross-linker *p*Bpa, the cross-links were absent without UV-irradiation. The *p*Bpa-dependence of cross-links to this position is already shown in Fig. 3. As additional control, we demonstrated that wild-type HiPIP *per se* does not give any shifts in the presence of *p*Bpa and UV (right blots). The presence of TatA, TatB, and TatC (pRK-*tatABC*) in the membranes and elution fractions was assessed as shown in Figs. 1 and 2. (C) HiPIP RR/KK variants with *p*Bpa (*) at indicated positions were produced in similar concentrations in the experiments shown in Fig. 1. Note significant precursor-accumulation in case of the KK-variants and more mature HiPIP in case of the RR-variants, indicating RR-dependent transport. Processing of KK-variants to mature size is mainly due to unspecific proteolytic degradation of the signal peptide. Detection in crude extracts with HiPIP specific antibodies.
(TIF)

S2 Fig Detectable Tat transport of HiPIP KK variants with optimized Tat motifs. Detection of HiPIP in subcellular fractions by SDS-PAGE/Western blotting, using HiPIP-specific antibodies. In control blots, purity of periplasmic and cytoplasmic fractions was confirmed by detection of periplasmic b-lactamase (bla) and cytoplasmic biotin carboxyl carrier protein (BCCP). Note some detectable transport of KK-HiPIP variants in which the Tat motif is optimized by an A13F or A13*p*Bpa substitution (*: mature periplasmic HiPIP). This position corresponds to the consensus “F” position in the motif, and F as well as *p*Bpa attribute a large, hydrophobic, aromatic side chain to the motif, which can promote the Tat translocon interaction and thus partially compensates for the transport-inactivating RR>KK exchange. Strains and conditions as in Fig. 1.
(TIF)

S3 Fig Determination of the density of TatA micelles found in the soluble fraction after cell disruption. (A) Experimentally determined densities of the fractions analyzed in B, showing identity of the two density profiles. (B) Detection of TatA and the polytopic membrane protein YidC (membrane marker) after isopycnic CsCl gradient centrifugation of soluble or membrane fractions from strain MC4100. No YidC is detectable in the soluble fraction, indicating that this fraction is virtually membrane-free. Note that soluble TatA micelles sediment to a density of ∼1.21 g/ml, which suggests a tight association with lipids but not with vesicles. Membrane-associated TatA sediments with membrane vesicles to a density of ∼1.18 g/ml, as confirmed by the detection of the membrane marker YidC (lower two blots).
(TIF)

S4 Fig Functionality and microscopic structure of TatA-*strep*. (A) Complementation of the chain formation phenotype of the Tat-deficient DADE strain by a single-copy chromosomally integrated *tatA-strep-tatBC* operon (strain DADE *tatA-strep-tatBC*). As controls, the Tat-system-containing parental strain MC4100 and the Tat-deficient strain DADE have been analyzed in parallel. (B) Tat transport of HiPIP as produced from pRK-*hip* in strain DADE *tatA-strep-tatBC* shown by Western-blot analysis of subcellular fractions. P: periplasm, M: membranes, C: cytoplasm. The periplasm contains transported mature (m) HiPIP, whereas only the cytoplasm contains unprocessed precursor (p) and some to mature size degraded HiPIP. The control blot on the right side detects the biotin carboxyl carrier protein (BCCP, cytoplasmic marker). (C) TatA micelles. Overview EM micrograph of purified TatA micelles from the cytoplasm of strain DADE *tatA-strep-tatBC*. The size bar indicates 100 nm.
(TIF)

S5 Fig Snapshot of a molecular dynamics simulation of TatA-HiPIP interaction. Color code: yellow: HiPIP signal peptide; magenta: HiPIP mature domain (FeS-cofactor in orange); red: TatA trans-membrane domain; green: TatA amphipathic helix.
(TIF)

S6 Fig HiPIP variants fold stably to completely cofactor-containing protein. Electronic absorption spectra of folded purified cofactor-containing HiPIP (wt) in comparison to the analyzed HiPIP variants with mutations in the mature domain (T50A, T50D, P104G, P104D, L119E). The spectra all show the typical [4Fe-4S]-cofactor absorption that indicates complete cofactor insertion and thus stable folding of HiPIP.
(TIF)

S1 Table Plasmids generated by QuikChange-mutagenesis and used primers. (DOCX)

S2 Table Plasmids based on cloning of standard PCR amplified fragments and used primers. (DOCX)
