# Supplementary figures and images for: TatBC-Independent TatA/Tat Substrate Interactions Contribute to Transport Efficiency
Source: PLoS One. 2015 Mar 16;10(3):e0119761. doi: 10.1371/journal.pone.0119761 (PMC4361764; doi:10.1371/journal.pone.0119761)

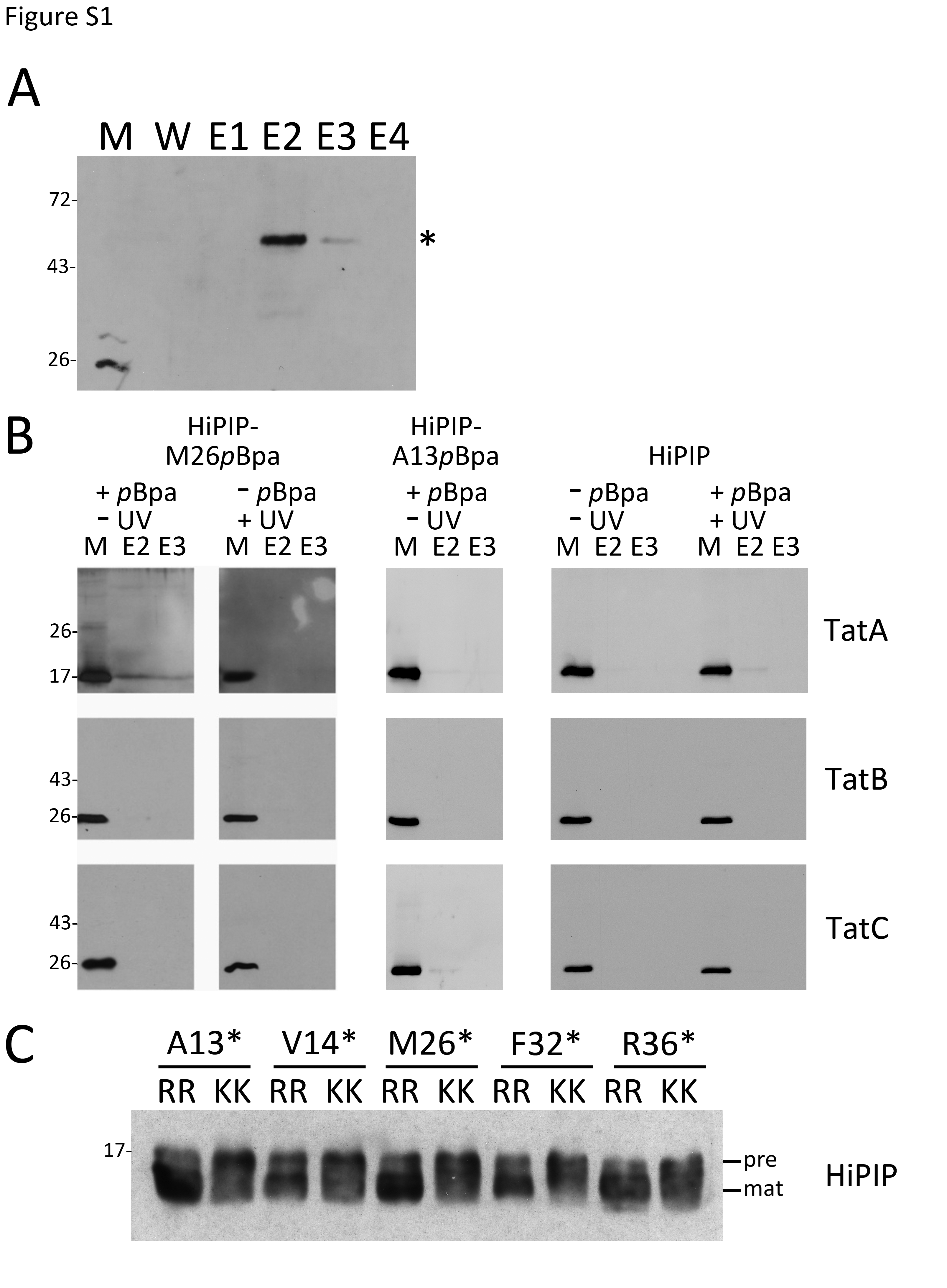

Supplement: S1 Fig — (A) During affinity chromatography, specific cross-links elute in fractions E2 and E3. Shown is the immunoblot-detection of cross-links of RR-HiPIP with a M26pBpa substitution to TatB, using antibodies recognizing TatB. M: solubilized membranes, W: last wash fraction, E1-E4: elution fractions. (B) Without UV irradiation or pBpa, no shifted bands can be detected in the elution fractions. For this important control, we chose HiPIP M26pBpa, which gives strong cross-links to all Tat components after UV irradiation (see Fig. 1A). No cross-links could be detected without UV irradiation or pBpa (+ pBpa/-UV, −pBpa/-UV, left blots). We also analyzed the UV-dependence of the cross-links for the HiPIP A13pBpa variant that was the most important variant in our studies. As expected for the UV-activatable cross-linker pBpa, the cross-links were absent without UV-irradiation. The pBpa-dependence of cross-links to this position is already shown in Fig. 3. As additional control, we demonstrated that wild-type HiPIP per se does not give any shifts in the presence of pBpa and UV (right blots). The presence of TatA, TatB, and TatC (pRK-tatABC) in the membranes and elution fractions was assessed as shown in Figs. 1 and 2. (C) HiPIP RR/KK variants with pBpa (*) at indicated positions were produced in similar concentrations in the experiments shown in Fig. 1. Note significant precursor-accumulation in case of the KK-variants and more mature HiPIP in case of the RR-variants, indicating RR-dependent transport. Processing of KK-variants to mature size is mainly due to unspecific proteolytic degradation of the signal peptide. Detection in crude extracts with HiPIP specific antibodies. (TIF) [file pone.0119761.s001.tif]

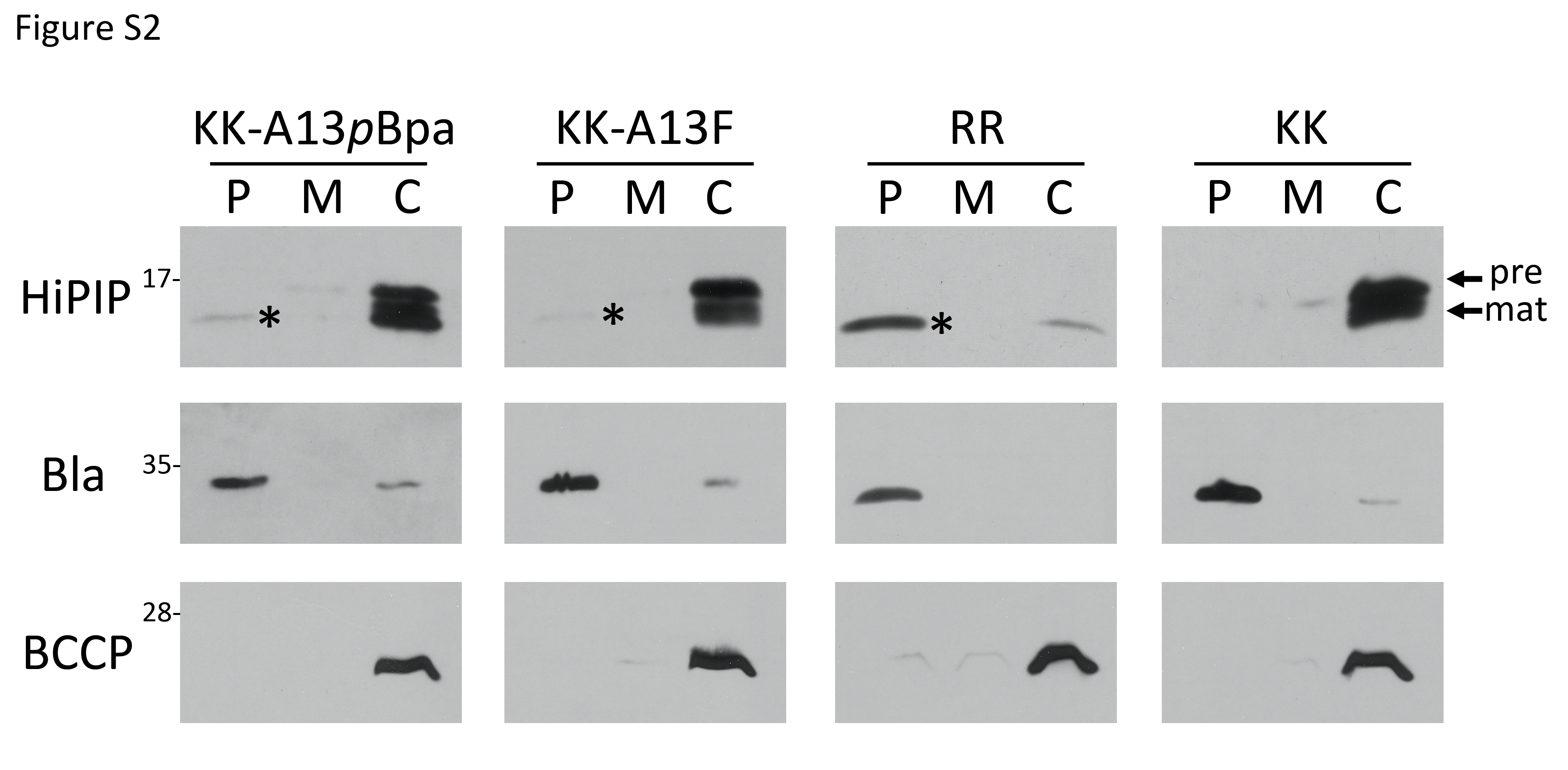

Supplement: S2 Fig — Detection of HiPIP in subcellular fractions by SDS-PAGE/Western blotting, using HiPIP-specific antibodies. In control blots, purity of periplasmic and cytoplasmic fractions was confirmed by detection of periplasmic b-lactamase (bla) and cytoplasmic biotin carboxyl carrier protein (BCCP). Note some detectable transport of KK-HiPIP variants in which the Tat motif is optimized by an A13F or A13pBpa substitution (*: mature periplasmic HiPIP). This position corresponds to the consensus “F” position in the motif, and F as well as pBpa attribute a large, hydrophobic, aromatic side chain to the motif, which can promote the Tat translocon interaction and thus partially compensates for the transport-inactivating RR>KK exchange. Strains and conditions as in Fig. 1. (TIF) [file pone.0119761.s002.tif]

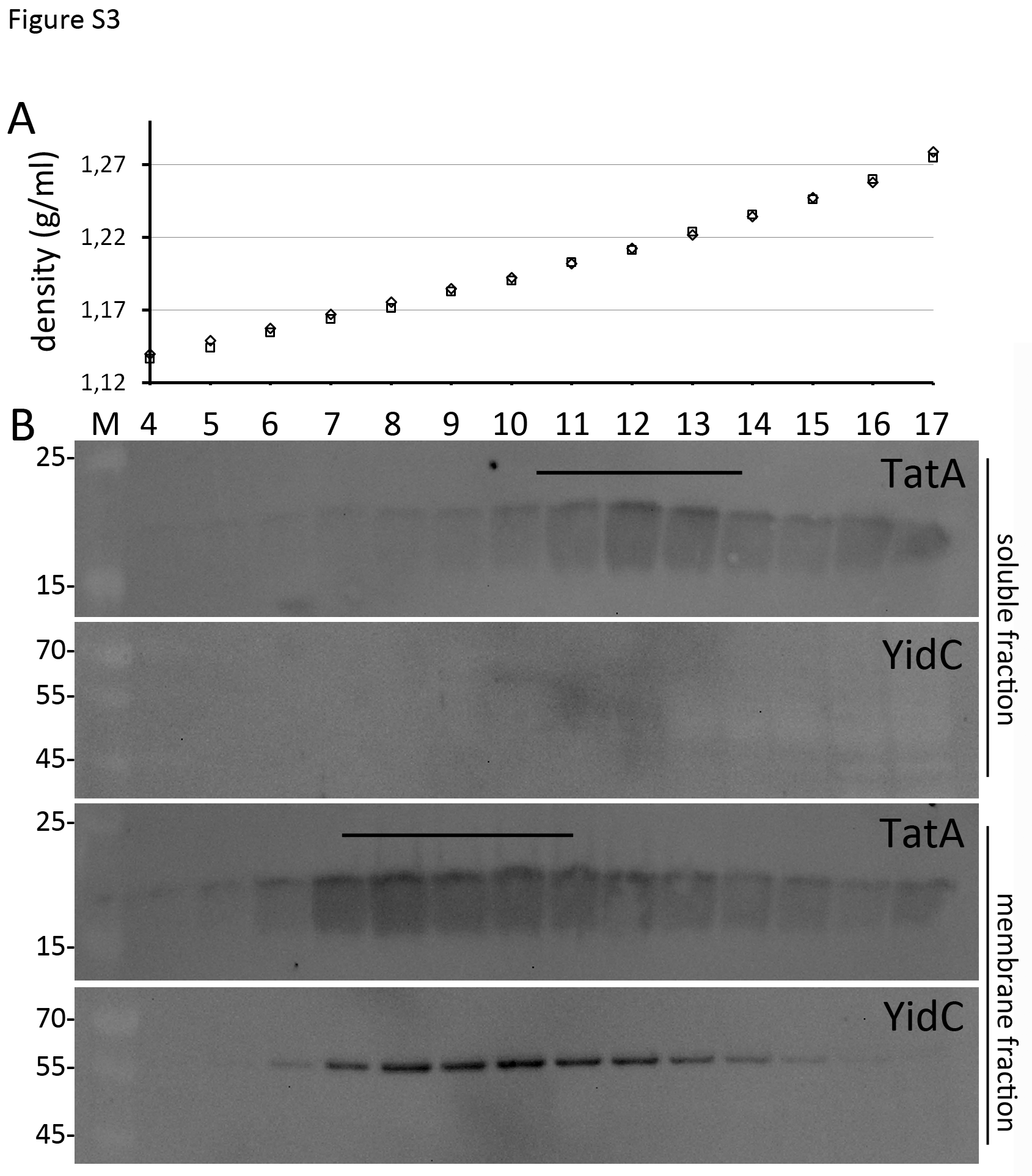

Supplement: S3 Fig — (A) Experimentally determined densities of the fractions analyzed in B, showing identity of the two density profiles. (B) Detection of TatA and the polytopic membrane protein YidC (membrane marker) after isopycnic CsCl gradient centrifugation of soluble or membrane fractions from strain MC4100. No YidC is detectable in the soluble fraction, indicating that this fraction is virtually membrane-free. Note that soluble TatA micelles sediment to a density of ∼1.21 g/ml, which suggests a tight association with lipids but not with vesicles. Membrane-associated TatA sediments with membrane vesicles to a density of ∼1.18 g/ml, as confirmed by the detection of the membrane marker YidC (lower two blots). (TIF) [file pone.0119761.s003.tif]

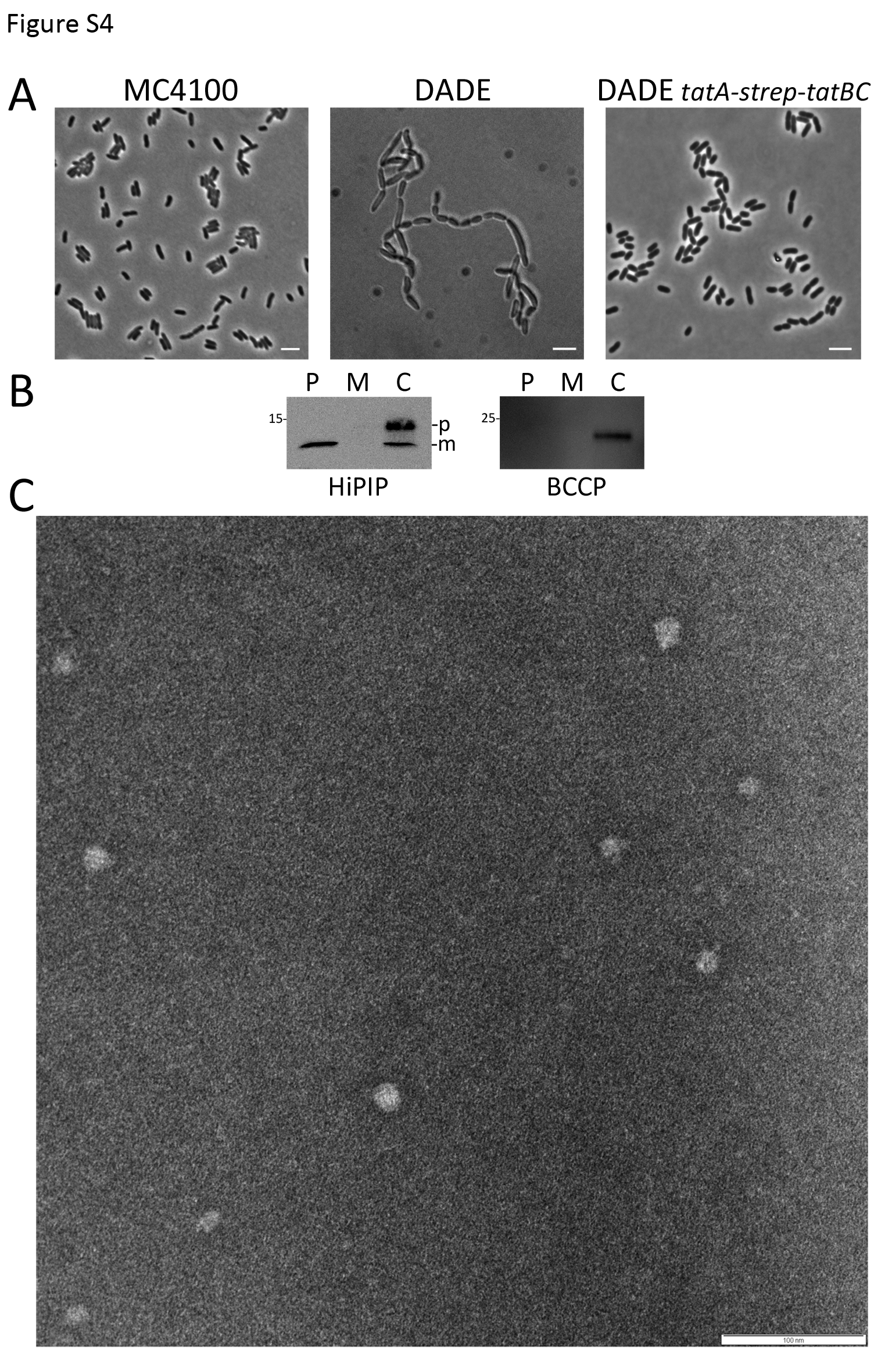

Supplement: S4 Fig — (A) Complementation of the chain formation phenotype of the Tat-deficient DADE strain by a single-copy chromosomally integrated tatA-strep-tatBC operon (strain DADE tatA-strep-tatBC). As controls, the Tat-system-containing parental strain MC4100 and the Tat-deficient strain DADE have been analyzed in parallel. (B) Tat transport of HiPIP as produced from pRK-hip in strain DADE tatA-strep-tatBC shown by Western-blot analysis of subcellular fractions. P: periplasm, M: membranes, C: cytoplasm. The periplasm contains transported mature (m) HiPIP, whereas only the cytoplasm contains unprocessed precursor (p) and some to mature size degraded HiPIP. The control blot on the right side detects the biotin carboxyl carrier protein (BCCP, cytoplasmic marker). (C) TatA micelles. Overview EM micrograph of purified TatA micelles from the cytoplasm of strain DADE tatA-strep-tatBC. The size bar indicates 100 nm. (TIF) [file pone.0119761.s004.tif]

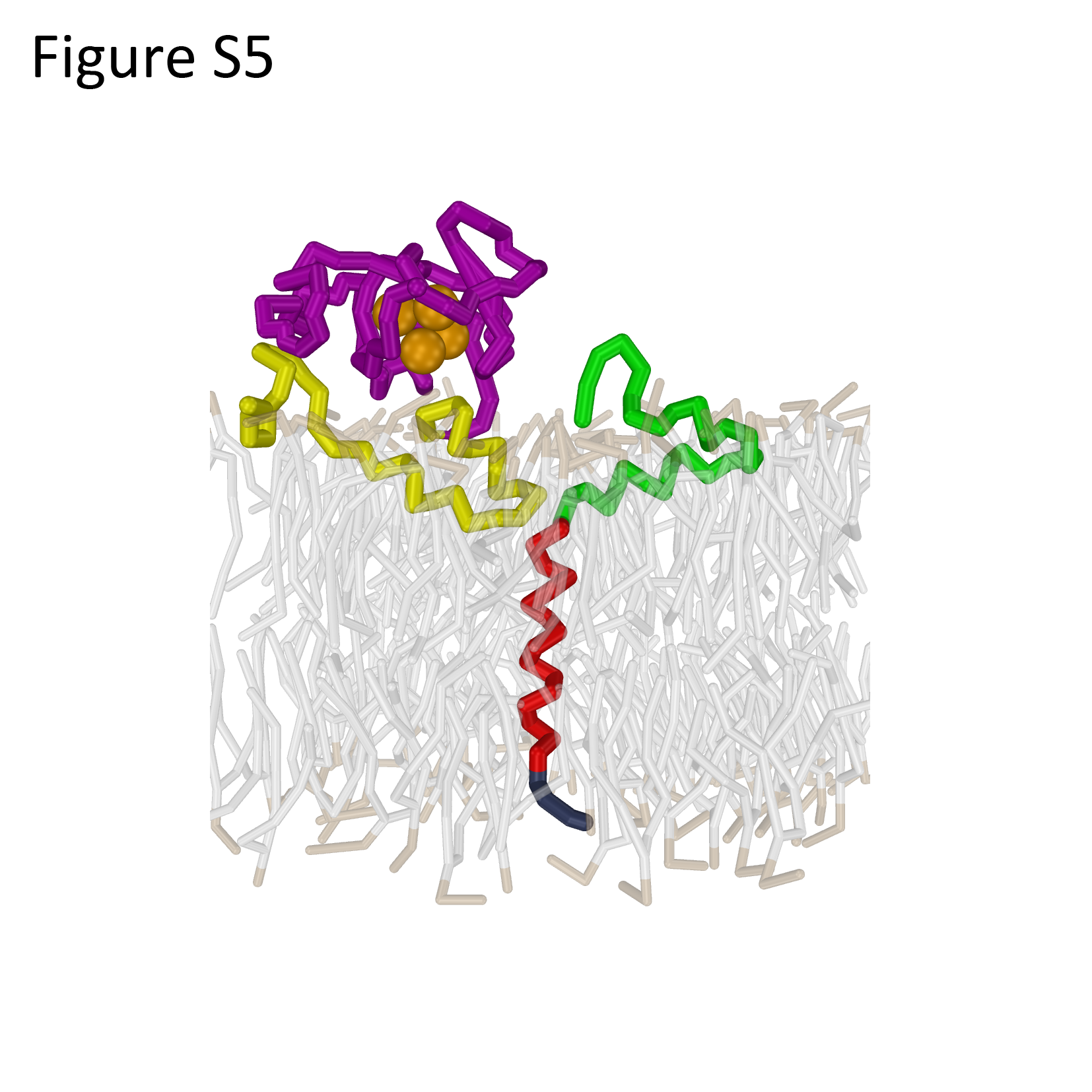

Supplement: S5 Fig — Color code: yellow: HiPIP signal peptide; magenta: HiPIP mature domain (FeS-cofactor in orange); red: TatA trans-membrane domain; green: TatA amphipathic helix. (TIF) [file pone.0119761.s005.tif]

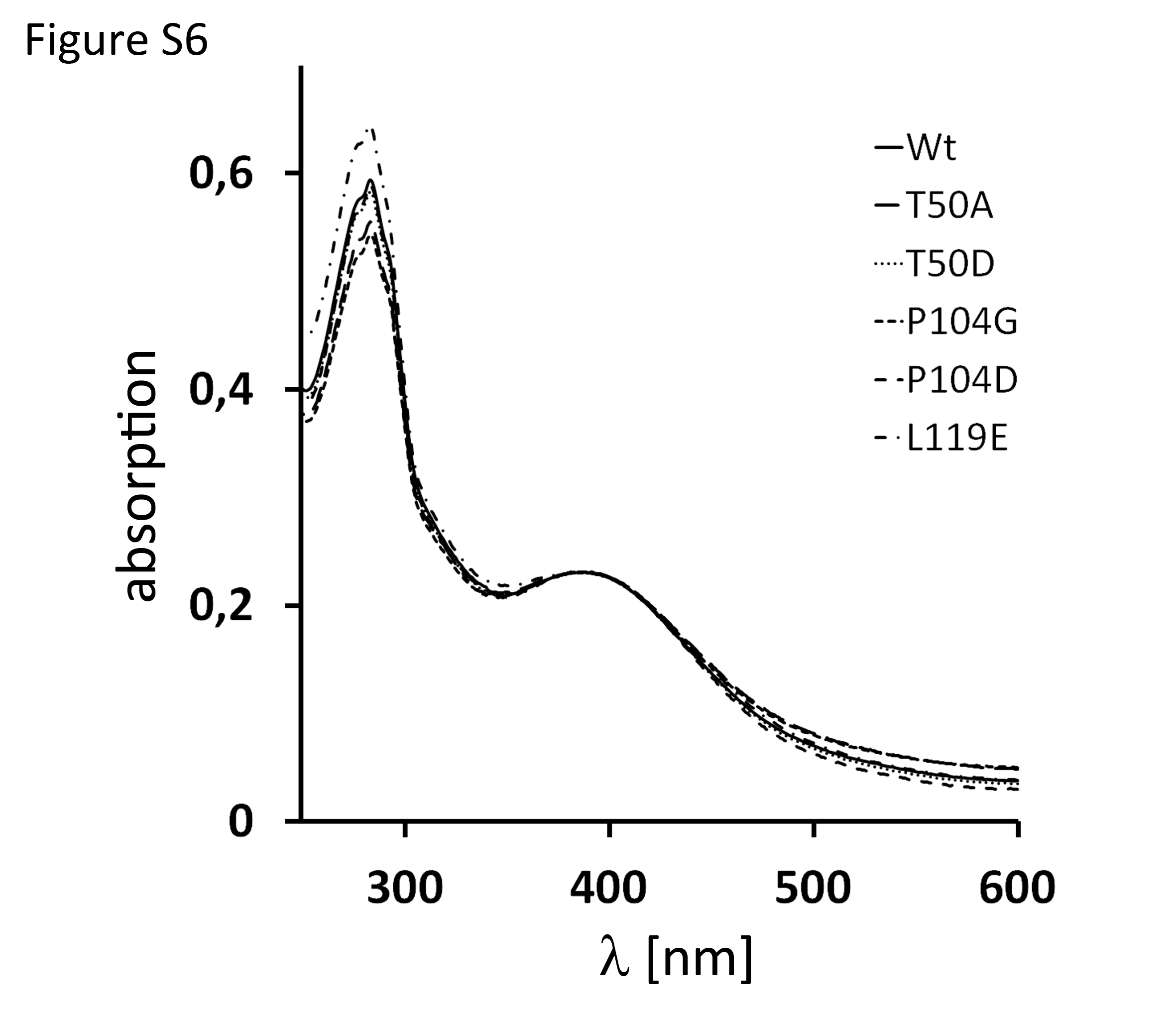

Supplement: S6 Fig — Electronic absorption spectra of folded purified cofactor-containing HiPIP (wt) in comparison to the analyzed HiPIP variants with mutations in the mature domain (T50A, T50D, P104G, P104D, L119E). The spectra all show the typical [4Fe-4S]-cofactor absorption that indicates complete cofactor insertion and thus stable folding of HiPIP. (TIF) [file pone.0119761.s006.tif]
