# Supplementary material for: TatBC-Independent TatA/Tat Substrate Interactions Contribute to Transport Efficiency
Source: PLoS One. 2015 Mar 16;10(3):e0119761. doi: 10.1371/journal.pone.0119761 (PMC4361764; doi:10.1371/journal.pone.0119761)
Supplement: S1 Table — (DOCX) [file pone.0119761.s007.docx]

**S1 Table: Plasmids generated by QuikChange-mutagenesis and used primers^1^**

| Plasmid | Primer | sequence 5´ 🡪 3´ |
| --- | --- | --- |
| pEXH5*tac*-RR-H6-A13*p*Bpa | *hip*-RR-A13*p*Bpa-F | AGC AAG AGC CGT CGT GAC TAG GTC AAA GTG ATG CTG |
| pEXH5*tac*-KK-H6-A13*p*Bpa | *hip*-KK-A13*p*Bpa-F | AGC AAG AGC AAG AAA GAC TAG GTC AAA GTG ATG CTG |
| pEXH5*tac*-RR-H6-V14*p*Bpa | *hip*-RR-V14*p*Bpa | AGC CGT CGT GAC GCT TAG AAA GTG ATG CTG GGC |
| pEXH5*tac*-KK-H6-V14*p*Bpa | *hip*-KK-V14*p*Bpa-F | AGC AAG AAA GAC GCT TAG AAA GTG ATG CTG GGC |
| pEXH5tac-KK-H6-A13F | *hip*-KK-A13F-F | CAA GAG CAA GAA AGA CTT TGT CAA AGT GAT GCT G |
| pEXH5*tac*-RR-H6-M26*p*Bpa, pEXH5*tac*-KK-H6-M26*p*Bpa | *hip*-M26*p*Bpa-F | GCC GCC GCC ATC CCG TAG ATC AAC CTG GTC GGT |
| pEXH5*tac*-RR-H6-F32*p*Bpa, pEXH5*tac*-KK-H6-F32*p*Bpa | *hip*-F32*p*Bpa-F | ATC AAC CTG GTC GGT TAG GGC ACC GCC CGT GCC |
| pEXH5*tac*-RR-H6-R36*p*Bpa, pEXH5*tac*-KK-H6-R36*p*Bpa | *hip*-R36*p*Bpa-F | GGT TTC GGC ACC GCC TAG GCC TCC GCT CCC GCC |
| pEXH5*tac*-H6-R10*p*Bpa | *hip*-R10*p*Bpa-F | CCA ATC AGC AAG AGC TAG CGT GAC GCT GTC AAA G |
| pEXH5*tac*-H6-R11*p*Bpa | *hip*-R11*p*Bpa-F | CA ATC AGC AAG AGC CGT TAG GAC GCT GTC AAA GTG |
| pEXH5*tac*-H6-K10*p*Bpa | *hip*-K10*p*Bpa-F | CCA ATC AGC AAG AGC TAG AAA GAC GCT GTC AAA G |
| pEXH5*tac*-H6-K11*p*Bpa | *hip*-K11*p*Bpa-F | CA ATC AGC AAG AGC AAA TAG GAC GCT GTC AAA GTG |
| pEXH5*tac*-H6-T50A | *hip*-T50A-F | CCG CGG ACG ATG CGG CCG CGA TCG CCC TC |
| pEXH5*tac*-H6-T50D | *hip*-T50D-F | CCG CGG ACG ATG CGG ACG CGA TCG CCC TC |
| pEXH5*tac*-H6-P104G | *hip*-P104G-F | GGC TGC CAG CTC TTC GGC GGC AAG CTG ATC AAC |
| pEXH5*tac*-H6-P104D | *hip*-P104D-F | GGC TGC CAG CTC TTC GAC GGC AAG CTG ATC AAC |
| pEXH5tac-H6-P104D-A13*p*Bpa | *hip*-RR-A13*p*Bpa-F | AGC AAG AGC CGT CGT GAC TAG GTC AAA GTG ATG CTG |
| pEXH5tac-H6-P104D-I52*p*Bpa | *hip*-I52*p*Bpa-F | GAC GAT GCG ACC GCG TAG GCC CTC AAG TAC AAC |
| pAH120-P*_tat_*-*tatA*-*strep-tatBC* | *tatA*-Bam-s-ATG-B-F | AGG TGG GAT CCT GGA GCC ACC CGC AGT TCG AAA AAT AAG CAG GTG TAA TCC ATG TTT GAT ATC GGT TTT AGC GAA C |
|  | pABS-*tatC*-BglII-R | ATA TAG CGC GCT TAT TCT TCA GTT TTT TCG CTT TC |
| pEX-*malE*(sp)-hip-H6  (based on pEX-*malE*(sp)-*hip*-H6-I3*p*Bpa) | *malE*-*hip*-I3-rev-F | GAT ATA CAT ATG AAA ATA AAA ACA GGT GCA CGC |

^1^the shown forward primers were used in conjunction with reverse primers that covered the identical sequence region
