# Supplementary material for: TatBC-Independent TatA/Tat Substrate Interactions Contribute to Transport Efficiency
Source: PLoS One. 2015 Mar 16;10(3):e0119761. doi: 10.1371/journal.pone.0119761 (PMC4361764; doi:10.1371/journal.pone.0119761)
Supplement: S2 Table — (DOCX) [file pone.0119761.s008.docx]

**S2 Table: Plasmids based on cloning of standard PCR amplified fragments and used primers**

| Plasmid | Primer | sequence 5´ 🡪 3´ | description |
| --- | --- | --- | --- |
| pEX-*malE*(sp)-*hip*-H6-I3*p*Bpa | *malE*-I3Bpa-*hip*-NdeI-F | ACA ACC ATA TGA AAT AGA AAA CAG GTG CAC GCA TCC TCG C | Amplification of *malE*(sp)-*hip* and introduction of UAG stop codon with pBW-*malE*(sp)*-hip-strep* as template. The NdeI and HindIII digested fragment was cloned into corresponding sites of pEXH5*tac*-H6 |
|  | *hip*-HindIII-R | ACA ACA AGC TTC AGG GTC CAG GAA GCG CAC C |  |
| pEXH5*tac*-mat-H6 | *hip*-mat-Nde-F | ACA ACC ATA TGT CCG CTC CCG CCA ATG CCG TGG CCG | Amplification of HiPIP mature domain encoding sequence with pEXH5*tac*-H6 as template, restriction with NdeI and HindIII and ligation into corresponding sites of pEXH5*tac*-H6 |
| pEXH5*tac*-H6-L119E | *hip*-Nde-F | ATA TAC ATA TGT CCG ATA AGC CAA TCA GCA AGA G | Amplification of *hip* and introduction of GAG codon (E) at position 119 of precursor encoding sequence with pEXH5*tac*-H6 as template, restriction with NdeI and HindIII and ligation into corresponding sites of pEXH5*tac*-H6 |
|  | *hip*-L119E-Hind-R | GCC GCA AGC TTC TCG GTC CAG GAA GCG CAC CAG CC |  |
